# Metabolic Reprogramming in Urological Tumors: New Perspectives from Tumor Metabolic Phenotypes to Therapeutic Targets

**DOI:** 10.7150/ijbs.123647

**Published:** 2025-10-27

**Authors:** Zhuohang Li, Lin Yang, Weijia Li, Wenxue Huang, Cunzhen Ma, Boyuan Sun, Xunguo Yang, Haoxiang Xu, Zhibiao Li, Jie Zhao, Bisheng Cheng, Peng Wu

**Affiliations:** 1 Department of Urology, Nanfang Hospital, Southern Medical University, Guangzhou, Guangdong, China.; 2 NMPA Key Laboratory for Research and Evaluation of Drug Metabolism, Guangdong Provincial Key Laboratory of New Drug Screening, School of Pharmaceutical Sciences, Southern Medical University, Guangzhou, 510515, China.; 3 Department of Surgery, Division of Urology, Beth Israel Deaconess Medical Center, Harvard Medical School, Boston, MA, USA.

**Keywords:** urological tumors, metabolic reprogramming, metabolic targeted therapy, metabolic marker

## Abstract

Metabolic reprogramming is a hallmark of cancer, enabling tumor cells to sustain growth, evade immune surveillance, and resist therapy. Urological tumors, including prostate, bladder, and renal cancers, exhibit distinct metabolic phenotypes driven by their unique tumor microenvironments and oncogenic pathways. This review explores the emerging landscape of tumor metabolism in urological cancers, highlighting key metabolic pathways such as glycolysis, lipid metabolism, amino acid metabolism, and redox balance. We discuss how these pathways are intricately linked to tumor progression, therapeutic resistance, and immune evasion. Furthermore, we examine novel therapeutic strategies targeting metabolic vulnerabilities, including metabolic enzyme inhibitors, synthetic lethality approaches, and metabolic modulation to enhance immunotherapy. By integrating advances in multi-omics technologies and preclinical models, we propose a framework for translating metabolic research into clinical applications. This review aims to provide a comprehensive overview of metabolic reprogramming in urological tumors and to identify potential metabolic targets for innovative therapies.

## 1. Introduction

Urological tumors, encompassing bladder cancer, renal cell carcinoma (RCC), and prostate cancer, are significant contributors to global cancer morbidity and mortality. Bladder cancer, which is associated with smoking and environmental exposure, is the 10th most common malignancy worldwide 1-3, with distinct molecular subtypes ranging from non-muscle-invasive to muscle-invasive disease 4. Despite advancements in surgical and systemic therapies, the five-year survival rates for advanced bladder cancer remain dismal. RCC, accounting for 80-85% of kidney cancers, is characterized by marked metabolic heterogeneity 5, with clear cell RCC (ccRCC) demonstrating unique metabolic hallmarks such as lipid accumulation and disruptions in oxidative phosphorylation 6, 7. Prostate cancer, the second leading cause of cancer-related deaths among men, displays remarkable clinical and molecular diversity 8-12, from hormone-sensitive localized disease to therapy-resistant, aggressive variants like neuroendocrine prostate cancer (NEPC) 13. While current therapies, including immune checkpoint inhibitors (ICIs) and androgen deprivation therapy, have improved outcomes, advanced disease stages continue to exhibit therapeutic resistance and poor prognosis 14-16.

Under specific physiological or pathological conditions, cells adjust their metabolism to meet their own needs, such as glycometabolism, lipid metabolism, and amino acid metabolism. But tumors exhibit significant changes in metabolic characteristics, which is called tumor metabolic reprogramming. In order to fight against different environments, tumor cells use the body's anabolism, such as the Warburg effect, lipid metabolism, and glutamine breakdown, to promote their own unrestricted growth. Such metabolic reprogramming not only supports tumor growth and metastasis but also provides the possibility of new therapeutic targets 17. Research on metabolic reprogramming in urologic tumors has gradually revealed some specific metabolic pathways and molecular mechanisms. For example, bladder cancer cells often exhibit significant enhancement of glycolysis 18-20, whereas renal cancer cells may maintain their growth and survival by altering lipid metabolism 21, 22. In addition, the metabolic profile of prostate cancer has also shown dependence on androgen-dependent metabolic pathways 23-25, and these findings suggest new targets and strategies for the treatment of urologic tumors.

This review aims to provide a comprehensive synthesis of recent advances in the field of metabolic reprogramming in urological tumors, focusing on bladder cancer, kidney cancer, and prostate cancer. We will explore the metabolic characteristics of these tumors, elucidate the mechanisms underlying metabolic reprogramming, and evaluate the clinical implications of these findings. By integrating multi-omics data—including genomics, transcriptomics, and metabolomics—this review seeks to uncover novel metabolic pathways and their regulatory networks. Additionally, we will discuss the therapeutic potential of targeting tumor metabolism, highlighting emerging strategies such as metabolic enzyme inhibitors, synthetic lethality approaches, and metabolic modulation to enhance immunotherapy. This review adopts a holistic perspective that encompasses both intrinsic tumor metabolic reprogramming and its interplay with the tumor microenvironment (TME), offering a roadmap for future research and clinical applications. Ultimately, this work aims to provide actionable insights into how metabolic vulnerabilities can be exploited to develop innovative therapies for urological malignancies.

## 2. Metabolic features of urologic tumors

### 2.1 Overview of tumor metabolism

#### Warburg effect and tumor metabolism

Metabolic reprogramming is a defining feature of cancer, enabling tumor cells to sustain their growth and proliferation by reconfiguring energy metabolism 26. One of the most well-known phenomena is the Warburg effect, characterized by a preference for aerobic glycolysis over mitochondrial oxidative phosphorylation, even under normoxic conditions 27. While glycolysis is less efficient in ATP production, it supports anabolic processes by generating intermediates essential for synthesizing nucleotides, lipids, and amino acids. This adaptation allows tumor cells to meet the biosynthetic and energetic demands of rapid proliferation 28. In urological tumors, such as bladder cancer, renal cell carcinoma (RCC), and prostate cancer, the Warburg effect plays a critical role in tumor progression. For example, in RCC, the hypoxia-inducible factor 1-alpha (HIF-1α) pathway drives the upregulation of glycolytic enzymes, including lactate dehydrogenase A (LDHA) and pyruvate kinase M2 (PKM2), enhancing glycolytic flux 29. Moreover, prostate cancer cells often exhibit AR-dependent modulation of glucose metabolism, with the androgen receptor (AR) promoting glucose transporter 1 (GLUT1) expression to increase glucose uptake. Additionally, the Warburg effect leads to the accumulation of lactate in the TME, creating an acidic and hypoxic milieu that promotes immune evasion and angiogenesis. High levels of lactate also disrupt normal metabolic signaling, reprogramming immune cells such as T cells and macrophages to adopt immunosuppressive phenotypes. Understanding the regulatory nodes of the Warburg effect, such as HIF-1α and LDHA, may provide new avenues for therapeutic interventions aimed at halting tumor progression and restoring immune function 17, 30-32.

#### Regulation of amino acid, lipid, and glucose metabolism

Amino acids play diverse roles in tumor metabolism, acting as substrates for protein synthesis, signaling mediators, and regulators of redox homeostasis. Among these factors, glutamine metabolism is a cornerstone of tumor growth. Glutamine serves as a nitrogen and carbon donor, fuelling the tricarboxylic acid cycle (TCA cycle), nucleotide biosynthesis, and antioxidant defense 33. Hypoxia and oncogenic signaling, such as c-Myc activation, upregulate glutaminase (GLS) to convert glutamine into glutamate, which feeds into the TCA cycle as α-ketoglutarate (α-KG). Glutamine-derived intermediates also support glucose-independent TCA activity, a critical adaptation in nutrient-limited environments 34-37. Other amino acids, such as serine and glycine, contribute to one-carbon metabolism and nucleotide synthesis, whereas arginine and polyamines promote tumor proliferation by modulating chromatin structure and transcriptional activity. Notably, polyamines also enhance nuclear factor kappa B (NF-κB) signaling, further driving tumor cell proliferation and invasion 33, 38, 39. These pathways represent promising therapeutic targets, particularly in tumors like RCC, where amino acid metabolism plays a central role in maintaining cellular homeostasis.

Reprogramming of lipid metabolism is essential for tumor progression. Tumor cells upregulate de novo fatty acid synthesis and enhance lipid uptake to meet the demands of membrane biosynthesis, energy production, and signaling. Lipids such as diacylglycerol (DAG) and phosphatidylinositol-3,4,5-triphosphate (PIP3) serve as second messengers, activating pathways involved in cell proliferation, angiogenesis, and metastasis. In prostate cancer, lipids also modulate AR signaling, reinforcing the metabolic dependency of these tumors on androgen-regulated lipid synthesis 40, 41. Alterations in lipid saturation levels protect cancer cells from reactive oxygen species (ROS)-induced damage. By increasing the ratio of saturated and monounsaturated fatty acids in membrane lipids, tumor cells enhance their resistance to oxidative stress, thereby supporting survival and therapy resistance 42. Targeting lipid metabolism, through inhibitors of fatty acid synthase (FASN) or lipid transporters, is an emerging strategy in the treatment of aggressive urological tumors.

Glucose is the primary energy source in mammalian cells, and tumor cells exploit its metabolism to fuel growth and survival. In addition to the Warburg effect, enhanced glucose uptake via GLUT transporters and increased glycolytic enzyme activity are hallmarks of tumorigenesis 43. Growth factor signaling, such as the *RTK/PI3K/AKT/mTOR* pathway, further amplifies glucose metabolism in tumor cells. Notably, the genomic amplification of receptor tyrosine kinases (e.g., EGFR, ERBB2) enhances the sensitivity of tumor cells to growth factors, sustaining their proliferative capacity 44. Tumor cells also influence stromal cells within the TME to secrete growth factors, creating a paracrine loop that supports tumor growth. For instance, bladder cancer cells can stimulate fibroblasts to secrete insulin-like growth factor (IGF), which activates downstream glucose metabolic pathways in both tumor and stromal cells 45. This reciprocal interaction underscores the complexity of metabolic reprogramming and its dependence on the TME.

#### Metabolic effects of TME

TME is mainly composed of tumor cells, immune cells, extracellular matrix (ECM), related metabolites, etc. These components of the TME interact with each other to jointly affect tumor growth, invasion, metastasis, and response to treatment. To meet the needs of rapid proliferation and survival, tumors adjust metabolic pathways to compete with other cells for a large number of nutrients, such as glucose and amino acids. The high consumption of glucose leads to the depletion of glucose in the TME, which affects the metabolism and function of immune cells. Moreover, the Warburg effect of tumors can lead to the accumulation of lactic acid in the TME and eventually form a hypoxic and acidic microenvironment, which also has a corresponding impact on metabolism. For glucose metabolism, in addition to the Warburg effect, immune cells in TME, such as T cells and macrophages, also adjust their glucose metabolism pathways to adapt to environmental changes 46, 47. Tumor cells may meet their energy requirements by increasing the uptake and oxidation of fatty acids. Meanwhile, alterations in lipid metabolism can also affect the function of immune cells; for example, lipid accumulation in dendritic cells may reduce their antigen presentation capacity 48. Amino acid metabolism has a profound impact on tumor development and progression. Amino acids are not only the cornerstone of protein synthesis, but also involved in the regulation of tumor cell metabolism, signal transduction, REDOX balance, and epigenetic modifications. For example, glutamine affects tumor-associated macrophages (TAMs) polarization and T cell differentiation, thereby affecting their related immune effect 49, and arginine metabolism can affect the function of CD8+ T cells 50. In general, TME has an impact on glucose, amino acid, and lipid metabolism, which not only promotes metabolic reprogramming but also downregulates the function of immune cells in TME and promotes tumor immune escape.

### 2.2 Metabolic reprogramming of bladder cancer

#### Abnormal glucose metabolism in bladder cancer

Bladder cancer, predominantly urothelial bladder cancer (UBC), is a highly vascularized malignancy characterized by metabolic reprogramming, which plays a pivotal role in its progression 51, 52. The rapid proliferation of tumor cells leads to the development of a disorganized vascular network, creating nutrient-deprived and hypoxic tumor microenvironments. Hypoxia is a critical driver of metabolic reprogramming in bladder cancer, particularly in glucose metabolism 53. Tumor endothelial cells (ECs), under hypoxic conditions, exhibit enhanced glycolytic flux, evidenced by the upregulation of glycolytic enzymes such as hexokinase 2 (HK2), PKM2, and LDHA. These changes are essential for angiogenesis and tumor growth 33, 54, 55. Metabolomic studies have revealed that UDP-GlcNAc, the terminal product of the hexosamine biosynthetic pathway (HBP), is significantly elevated in ECs derived from muscle-invasive bladder cancer (MIBC) tissues 56, 57. These findings suggest that the key HBP enzyme, GFAT1, may drive glucose metabolism reprogramming in bladder cancer. Moreover, studies have shown that the overexpression of glucose metabolism-related proteins, such as GLUT1 and HK2, correlates with increased aggressiveness and poor prognosis in bladder cancer. Experimental inhibition of HK2 via 2-deoxy-D-glucose (2-DG) has demonstrated promising antitumor effects *in vitro* and *in vivo*, indicating the potential therapeutic value of targeting glucose metabolism in bladder cancer 58, 59. Studies have evaluated the association between GLUT1 and the 10-year overall survival (OS) of patients. Compared with tumors with low expression, patients with tumors overexpressing GLUT1 had a lower 10-year OS (97 months vs. 163 months, log rank P = 0.004) 60.

Resistance to chemotherapeutic agents such as cisplatin and gemcitabine is another major challenge in bladder cancer management 61. Hypoxia in the TME promotes metabolic adaptations that contribute to chemoresistance, including increased glycolytic flux through the pentose phosphate pathway (PPP) and increased synthesis of deoxycytidine triphosphate (dCTP), which competes with gemcitabine for DNA incorporation. Furthermore, glucose-derived acetate and acetyl-CoA synthetase 2 (ACSS2) have been implicated in cisplatin resistance. Elevated glucose consumption in cisplatin-resistant cells leads to higher production of glucose-derived acetate, fueling fatty acid synthesis and promoting tumor survival. Targeting ACSS2 and other metabolic vulnerabilities represents a promising strategy for overcoming chemoresistance in bladder cancer 62-66. Metabolomic analysis has shown that metabolic reprogramming in gemcitabine-resistant urothelial cancer cells increases aerobic glycolysis via the pentose phosphate pathway, promotes the synthesis of dCTP, and competitively inhibits gemcitabine 67. Glucose-derived acetate and ACSS2 are also factors involved in cisplatin resistance in bladder cancer. In cisplatin-resistant bladder cancer cells (T24R cell line), glucose consumption is increased, leading to higher production of glucose-derived acetate and fatty acids. ACSS2 provides acetyl-CoA to tumor cells via glucose-derived endogenous acetate in these cells and is a potential target for cisplatin resistance 68. Glucose metabolic reprogramming in bladder cancer plays an important role in tumor growth, angiogenesis, and chemoresistance, especially in a hypoxic environment. Targeting glucose metabolism and metabolic vulnerabilities, such as gfat1 and acss2, may play a potential role in delaying bladder cancer tumor progression and improving chemotherapy resistance.

#### Lipid metabolism in bladder cancer and its clinical implications

Reprogramming of lipid metabolism is one of the most prominent metabolic changes in bladder cancer. The changes of lipid metabolism include the characteristic changes of key genes and metabolic components, such as lipid uptake, synthesis, transport, and catabolism, to adapt to the growth needs of tumor cells. A mixed cohort study of 800,000 people suggested positive associations between blood pressure, cholesterol, triglycerides, and risk for NMIBC and bladder cancer mortality in men and between triglycerides and bladder cancer mortality in women 69. Metabolomic analysis further indicates that bladder cancer tissue exhibits elevated levels of phospholipids and fatty acids while demonstrating lower triglycerides compared to normal bladder tissue 70. Blocking or inhibiting lipid metabolism-related proteins, such as peroxisome proliferator-activated receptors (PPARs), Sterol-regulatory element binding proteins (SREBPs), and FASN, can inhibit the proliferation and metastasis of bladder cancer cells 71, 72. In addition, HSDL2, FADS1, FATP4, ACSL1, and other proteins, which are closely related to the proliferation and apoptosis of bladder cancer cells, are highly expressed in bladder cancer tissues 73, 74.

Aberrant activation of SREBP is the main cause of abnormal lipid metabolism in bladder cancer. SREBP1 is mainly involved in fatty acid synthesis, whereas SREBP2 mainly regulates cholesterol biosynthesis 75. Activating mutations in the RAS gene are associated with lipid metabolism remodeling, which can activate mammalian target of rapamycin C1 (mTORC1), which in turn induces mature SREBP to enter the nucleus and promote the transcription of genes related to lipid metabolism. To meet the needs of tumor cell growth and metabolism 76, 77. SREBP plays important roles in lipid metabolism, tumor growth, tumor stemness maintenance, and chemotherapy resistance in bladder cancer, which may be a potential target for the treatment of bladder cancer.

High cholesterol not only increases the prevalence of cardiovascular diseases, but is also closely related to bladder cancer. Excess cholesterol in serum can increase the tumor dryness of transplanted bladder cancer mice and spontaneous bladder cancer, thus promoting the development of bladder cancer. This is closely related to Oxidized low-density lipoprotein (ox-LDL) in serum 78. For patients with hypercholesterolemic bladder cancer, serum ox-LDL can bind to CD36 and activate the *JAK2-pSTAT3* pathway, thereby promoting the cancer stemness of bladder cancer, increasing cancer cell proliferation, and inducing epithelial-mesenchymal transition *in vitro*. In addition to cancer cells, ox-LDL can interact with other cells through the CD36 receptor, interact with macrophages to secrete proinflammatory cytokines and chemokines, and interact with endothelial cells to promote angiogenesis 79, 80. Therefore, the level of ox-LDL in serum can predict the prognosis of patients to some extent. Targeting cholesterol metabolism is one of the feasible methods in treatment strategies. 7-dehydrocholesterol reductase (DHCR7) is an important enzyme involved in cholesterol synthesis. It can promote the *cAMP/PKA/AKT* pathway by reducing the concentration of 7-dehydrocholesterol and promoting the transcription of G protein-coupled receptor, which plays an important role in bladder cancer invasion and metastasis. Inhibition of DHCR7 is expected to be a feasible therapeutic strategy to inhibit bladder cancer invasion and metastasis 81.

Apart from glucose metabolism, fatty acid β-oxidation also provides energy to tumor cells. Therefore, the key enzymes of lipid metabolism are expected to be potential targets for the treatment or adjuvant therapy of bladder cancer. Studies have shown that FASN is one of the core genes of fatty acid metabolism in bladder cancer and is related to the efficacy of immune checkpoint therapy in bladder cancer. Patients with low expression of FASN have a better response to ICI treatment. In turn, it is suggested that FASN is also a potential indicator and regulator of ICI therapy 82. Through integrated bioinformatics analysis, researchers can use genes that are key to lipid metabolism to construct diagnostic and therapeutic models of bladder cancer and develop novel gene signatures related to lipid metabolism that can predict the prognosis of patients with bladder cancer and may guide treatment selection 83, 84.

In conclusion, lipid and cholesterol metabolism reprogramming plays a crucial role in the occurrence and development of bladder cancer, providing a new perspective and potential therapeutic target for the diagnosis, treatment, and prognosis evaluation of bladder cancer.

#### Metabolic markers associated with bladder cancer progression

Urine cytology is one of the important methods for the diagnosis and postoperative follow-up of bladder cancer. There may be changes in specific metabolites in the urine of patients with bladder cancer. At present, urine fluorescence *in situ* hybridization (FISH), nuclear matrix protein 22 (NMP22), bladder tumor antigen (BTA), and other detection methods are available. Urinary NMP22 and BTA can be significantly increased in patients with bladder cancer, which is of reference value for the diagnosis of bladder cancer 85. Techniques for detecting bladder cancer based on urine DNA or RNA, such as the detection of mutations and methylation of telomerase reverse transcriptase (TERT), Fibroblast growth factor receptor 3 (FGFR3), Vimentin (VIM), and One Cut Homeobox 2 (ONECUT2) genes, have become the focus of research. These gene mutations and methylation signatures have high sensitivity and specificity for the diagnosis of bladder cancer 86-89. Detection of the mRNA expression of* IGFBP5, HOXA13, MDK, CDC2, CXCR2*, etc., is also helpful for the early diagnosis of bladder cancer 90, 91. According to the results of weighted gene co-expression network analysis and protein-protein interaction network analysis, some researchers have identified 6 potential biomarkers (COL3A1, FN1, COL5A1, FBN1, COL6A1, and THBS2) that may be related to the progression and poor prognosis of bladder cancer, but further studies are lacking.

Urine examination provides a variety of detection methods for the diagnosis and postoperative follow-up of bladder cancer, including traditional urine cytology and the detection of emerging molecular markers. These methods help improve the diagnostic accuracy and prognostic evaluation of early bladder cancer.

### 2.3 Metabolic reprogramming of renal cell carcinoma

#### Metabolic features of clear-cell renal cell carcinoma: lipid accumulation and oxidative phosphorylation

ccRCC is the most common pathological type of renal cancer. It is typically characterized by the deposition of cytoplasmic lipids, which are necessary to maintain cell survival, but excess lipids can also promote renal tumor invasion 92. In the fatty acid synthesis pathway, enzymes such as FASN, ATP citrate lyase (ACLY), and acetyl-CoA carboxylase (ACC) are up-regulated in ccRCC cancer tissues 93. Interestingly, increased expression of ACLY is associated with a good prognosis for patients. However, *in vitro* cell experiments have shown that tumor cell proliferation is reduced when ACLY is inhibited, and the association between this needs to be further explored 94. High expression of ACC and FASN is associated with poor prognosis of patients (Hazard ratio (HR) 5.563, 95% confidence interval (CI) = 3.431 - 9.021, P < 0.001) 95. A new generation FASN inhibitor, TVB-2640, has shown promising effects in phase I trials in patients with advanced cancer (including prostate cancer, rectal cancer, gastric cancer, etc.

), suggesting the feasibility of FASN as a therapeutic target 96.

CD36, a transmembrane protein that transfers extracellular fatty acids to cytoplasmic fatty acid binding proteins, is regulated by the HIF pathway, which could explain its upregulation in renal cancer 97. In addition to renal cell carcinoma, CD36 expression is also upregulated in many malignancies, including prostate cancer, and in patients undergoing nephrectomy, progression-free survival (PFS) is significantly reduced in patients with high CD36 expression, which is dependent on exogenous intake of cholesterol. Among the receptors related to cholesterol intake, the transporter scavenger receptor-B1 (SCARB1) was significantly upregulated. These results indicate that ccRCC tumors mainly rely on SCARB1 for cholesterol intake. However, studies have shown that cholesterol promotes tumor cell proliferation mainly through the *PI3K/AKT* signaling pathway, while SCARB1-mediated cholesterol uptake also protects tumor cells from ROS-mediated oxidative stress 98, 99.

In ccRCC, the Warburg effect is also present, where the oxidative phosphorylation process is usually inhibited and the glycolytic pathway is upregulated. HIF inhibits the TCA cycle through transcriptional activation of pyruvate dehydrogenase kinase 1(PDK1). Most enzymes that catalyze the TCA cycle in renal cancer cells are down-regulated, and mitochondrial oxidative phosphorylation activity, which is closely related to TCA, is also reduced. Other enzymes whose pathways supplement metabolic flow to the TCA cycle are also commonly downregulated 100, 101.

In ccRCC, mitochondrial electron transport chain (ETC) activity is decreased, but TCA cycle activity is increased in metastatic ccRCC. Stimulation of mitochondrial respiration or activation of the NADH cycle promotes tumor metastasis, while inhibition of electron transport chain complex I activity inhibits metastasis. In addition, higher expression of genes involved in oxidative phosphorylation is associated with the aggressiveness of ccRCC, and higher mtDNA content is also associated with poorer survival expectations 102.

The oxidative phosphorylation process in ccRCC is also associated with oxidative stress. For example, DPP9 induces ccRCC resistance to targeted therapy by stabilizing NRF2 protein levels, activating oxidative stress signaling, and inhibiting ferroptosis. NRF2 has a wide range of antioxidant effects and is a key gene in the regulation of oxidative stress. In ccRCC, NRF2 activation is clearly associated with tumor progression and drug resistance. These research advances reveal the critical role of lipid accumulation and oxidative phosphorylation in ccRCC in tumor metabolic reprogramming, and suggest a dual role of oxidative phosphorylation in the progression of renal cell carcinoma 103.

Lipid accumulation and oxidative phosphorylation play a central role in the metabolic reprogramming of ccrcc. Among them, the role of OXPHOS is dual (inhibited in primary tumors and promoted in metastasis) and closely associated with other processes (such as oxidative stress and drug resistance), revealing its complexity as a therapeutic target.

#### Lactate metabolism and microenvironmental adaptation in renal cell carcinoma

The metabolic reprogramming of RCC involves significant alterations in lactate metabolism, which plays a critical role in shaping TME and driving disease progression. Cancer-associated fibroblasts (CAFs) are key players in this process and actively participate in glycolysis and lactate secretion. This phenomenon, driven by the Warburg effect, contributes to hypoxia and decreased pH in the TME, fostering an environment conducive to tumor growth and immune evasion.

CAFs exhibit dual roles in the TME. On the one hand, they secrete growth factors and chemokines, attracting immune cells to the tumor site and producing anti-tumor effects. On the other hand, CAF-derived exosomes can transfer oncogenic molecules into renal cancer cells, promoting their proliferation, invasion, and metastatic potential.

The accumulation of lactate in the TME creates an immunosuppressive milieu, facilitating tumor progression through mechanisms such as polarization of TAMs toward the M2 phenotype, impaired dendritic cell differentiation, and suppression of cytotoxic T cell functions 104, 105. Recent studies highlight that lactate induces epigenetic modifications, such as histone lactylation in M1 macrophages, altering their phenotype and reducing their anti-tumor activity. Conversely, glutamine synthetase (GS) activity in M2 macrophages mediates their pro-angiogenic, immunosuppressive, and pro-metastatic functions. Targeting GS has been shown to reprogram M2 macrophages back into M1 macrophages, thereby enhancing T cell-mediated immune responses 106, 107. Thus, lactate metabolism plays a central role in RCC progression, not only by fuelling tumor cell growth but also by modulating immune cell function, enabling immune escape, and contributing to therapy resistance.

#### Role of HIF pathway in renal cell carcinoma metabolism

Metabolic reprogramming in RCC is heavily influenced by the inactivation of the *VHL (von Hippel-Lindau)* gene and the activation of the *Ras-PI3K-AKT-mTOR* pathway. Loss of VHL function leads to the stabilization and activation of HIFs, which are key transcription factors driving hypoxia-associated metabolic adaptations 108. RCC is characterized by high HIF activity, particularly involving HIF-1α and HIF-2α, which orchestrate distinct but complementary roles in tumor progression.

HIF-1α is a critical driver of the Warburg effect, promoting glycolysis through upregulation of glycolytic enzymes such as LDHA, GLUT1, and PDK1. These metabolic shifts increase lactate production, contributing to TME acidification and angiogenesis through the induction of vascular endothelial growth factor (VEGF) and platelet-derived growth factor (PDGF) 109. Additionally, HIF-1α-mediated lactate accumulation enhances ferroptosis resistance in RCC by modulating the pH-dependent HIF-1α/LDH axis, enabling tumor cells to survive oxidative stress 110.

HIF-2α, on the other hand, plays a prominent role in lipid metabolism and cancer stem cell (CSC) maintenance. Studies indicate that the HIF pathway is linked to lipid deposition in RCC, with PHF8, a regulator of lipid storage, being activated under hypoxia in a VHL-dependent manner 111. Furthermore, HIF-2α drives the expression of genes associated with invasion and stemness, such as *OCT4, PAI-1, and MMP9*, through the activation of hypoxia-associated factors (HAFs) that shift hypoxia responses from HIF-1α to HIF-2α dependency 110. The role of HIFs in CSC maintenance, while well-documented in other cancers, remains underexplored in RCC. Insights from glioblastoma suggest that HIF-2α, rather than HIF-1α, is more critical for CSC proliferation and self-renewal, highlighting the potential for similar mechanisms in RCC. Upregulated HIF-2α in CSCs has been associated with the promotion of tumor-initiating capacity, immune evasion, and therapeutic resistance 112, 113. Given the aggressive nature of CSC-driven RCC, further investigation into the interplay between HIFs and CSCs is warranted.

### 2.4 Metabolic reprogramming of prostate cancer

#### Abnormal lipid metabolism and androgen-receptor signaling in prostate cancer

Abnormal lipid metabolism is one of the significant characteristics of PCa, which is closely related to the development of the disease and treatment resistance. Prostate tumors are highly dependent on lipids for energy, growth, and survival. The androgen receptor is a major factor in the growth and progression of prostate cancer. It is a ligand-dependent nuclear transcription factor that regulates the expression of target genes by binding to androgens. Androgen deprivation therapy (ADT) is the cornerstone of the treatment of hormone-sensitive prostate cancer, but most patients will eventually develop castration-resistant prostate cancer (CRPC), where AR signaling remains active even in the presence of very low androgen levels.

AR directly regulates the expression of genes encoding proteins involved in lipid synthesis, uptake, and storage. AR can also indirectly affect key regulators of lipid metabolism by increasing the expression of sterol regulatory element binding proteins 114, 115. For example, it has been shown that AR can directly regulate* acyl-CoA synthetase medium-chain family members 1 and 3 (ACSM1 and ACSM3)*, both of which are up-regulated in tumor tissues. AR promotes tumor proliferation and protects tumor cell survival both *in vitro* and *in vivo*. The metabolic dysregulation caused by ACSM1/3 deficiency promotes ferroptosis in tumor cells 116.

SREBP is a major transcriptional regulator of lipogenesis; AR can control SREBP function by regulating SREBP cleavage—activating protein (SCAP) expression, and activation of SREBP promotes the transcription of lipogenic enzymes 117, 118. These findings explain the upregulated expression of genes related to lipid metabolism in PCa, including *FASN, LDLR, ACC, and phosphoenolpyruvate kinase 1 (PCK1).*

Fatty acid binding protein 5 (FABP5) is a key transporter that delivers fatty acids to nuclear receptors to enhance PCa metastasis 119. The signaling lipids produced by FABP5 and monoacylglycerolase (MAGL) enter the nucleus and bind to receptors such as PPARγ to regulate VEGF factor synthesis and promote tumor metastasis 120. In addition, AR signaling can interact with the *PTEN/PI3K/AKT/mTOR* pathway. In addition, the *PI3K/AKT/mTOR* pathway plays an important role in cell cycle regulation, and both of them jointly promote tumor proliferation. Dual pathway inhibition can significantly inhibit tumor growth 121. AR signaling can also interact with related transcription factors in the* WNT* signaling pathway to promote the activation of the *Wnt/β-catenin* signaling pathway 122.

AR reprograms lipid metabolism through a multi-level complex network (direct, indirect, and pathway intersection) to meet the bioenergetic and biosynthetic needs of prostate cancer cells, which not only drives tumor progression and metastasis, but also leads to the generation of therapeutic resistance (such as castration resistance). This makes targeting lipid metabolism a potential therapeutic strategy.

#### Glycometabolism in prostate cancer and its relationship with disease grade

PCa, like other malignancies, exhibits a metabolic shift toward aerobic glycolysis. Advanced PCa cells exhibit high glycolytic activity, and key enzymes of glycolysis have been confirmed to be associated with cancer. Among the GLUT subtypes, GLUT1 is closely related to PCa. It is frequently overexpressed in PCa and is associated with a poor prognosis of PCa^123^.

High expression of key enzymes in glycolysis, hexokinase (HK) and phosphofructokinase (PFK), is also responsible for tumor progression. ADP-dependent glucokinase (ADPGK) is overexpressed in prostate adenocarcinoma and has been shown to promote PCa cell proliferation and migration *in vitro* and *in vivo*, while predicting poor prognosis of patients 124. The end product of glycolysis, lactate, and the acidic TME it creates play critical roles in PCa metastasis. Accumulated lactate enhances tumor cell motility and invasiveness while suppressing immune surveillance, thus facilitating disease progression 31, 125, 126.

The metabolic landscape of PCa is further linked to tumor grade, as classified by the Gleason scoring system. This scoring system assesses tumor aggressiveness based on histological patterns. Emerging evidence indicates that glucose metabolism levels correlate with PCa grade and stage. For instance, studies suggest that hyperglycemia has distinct prognostic implications depending on tumor grade: Low-grade PCa: Elevated HbA1c levels (a marker of long-term blood glucose levels) appear to be negatively associated with disease progression. High-grade PCa: HbA1c is positively associated with tumor aggressiveness and poor prognosis.

These findings highlight a complex relationship between glucose metabolism, systemic hyperglycemia, and disease severity. Hyperglycemia may fuel glycolysis-dependent pathways in aggressive PCa, exacerbating tumor progression, while lower glucose availability may restrict growth in indolent tumors 127.

#### Role of Oxidative Stress in Prostate Cancer Progression

Oxidative stress arises from an imbalance between the production of ROS and the cellular antioxidant defense system. In prostate cancer, ADT and interference with AR signaling, the cornerstone treatments for advanced disease, profoundly alter redox homeostasis. Under normal conditions, androgens regulate the balance between pro-oxidants and antioxidants, maintaining physiological redox equilibrium in the prostate. However, following castration, ROS production increases significantly, disrupting this balance and enhancing oxidative stress 128.

Excess ROS plays a critical role in prostate cancer progression by acting as signaling molecules that regulate key oncogenic pathways. Oxidative stress activates transcription factors such as *Twist1, YB-1, NF-κB, and CREB,* which drive AR signaling. This activation leads to increased AR expression, heightened AR sensitivity to low androgen levels, and local production of androgens within TME. In addition, oxidative stress can induce alternative pathways of AR activation, further promoting castration resistance and tumor progression 129-132.

In patients with prostate cancer, there are changes in antioxidant defense capacity, such as increased plasma concentrations of thiobarbituric acid-reactive substances (TBARS), increased serum protein carbonylation, decreased whole blood catalase (CAT) activity, increased superoxide dismutase (SOD) activity, and increased plasma and red blood cell thiol levels. Serum vitamin C and vitamin E concentrations are reduced 133. HJURP enhances the antioxidant capacity of oxidase-1 (PRDX1) through disulfide bonds, leading to a novel mechanism of ferroptosis resistance in prostate cancer cells. This study provides potential new therapeutic targets for prostate cancer 134.

In summary, oxidative stress, driven by increased ROS production following androgen deprivation, plays a central role in prostate cancer progression by activating AR signaling pathways and inducing treatment resistance. Prostate cancer cells adapt by upregulating antioxidant mechanisms, including enzymatic systems and non-enzymatic antioxidants, to counterbalance ROS and evade ferroptosis. The interplay between oxidative stress, antioxidant defenses, and ferroptosis resistance not only drives disease progression but also offers novel opportunities for therapeutic intervention. Targeting these pathways could lead to improved outcomes for patients, particularly in advanced prostate cancer.

## 3. Molecular mechanisms of metabolic reprogramming

### 3.1 Key metabolic enzymes and signaling pathways

#### mTOR and PI3K/AKT pathways and metabolic regulation

The *mTOR* signaling pathway is a central regulator of cellular growth, proliferation, and metabolism. It functions through two distinct protein complexes: mTORC1 and mTORC2, each with unique regulatory roles. mTORC1 primarily controls anabolic processes, including protein synthesis, lipid synthesis, and glycolysis, while mTORC2 regulates cell survival, cytoskeletal organization, and lipid metabolism.

Dysregulation of the *PI3K/AKT/mTOR* axis is a hallmark of cancer metabolic reprogramming, contributing to uncontrolled cell growth and survival. mTORC1 is activated by upstream signals such as growth factors (e.g., insulin), amino acids (e.g., leucine), and energy status. Activation occurs through the* PI3K-AKT-TSC1/2-Rheb* cascade, where AKT phosphorylates TSC2 to relieve its inhibitory effects on Rheb, enabling mTORC1 activation 135.

Once activated, mTORC1 promotes glycolysis by inducing key transcription factors, including hypoxia-inducible factor 1α (HIF1α) and Myc, both of which upregulate glycolytic enzymes such as GLUT1, HK2, and LDHA. These enzymes increase glucose uptake and glycolytic flux, enabling cancer cells to meet the biosynthetic demands of rapid proliferation.

Additionally, mTORC2 regulates glycolysis via both AKT-dependent and AKT-independent mechanisms. AKT activation promotes glucose metabolism by phosphorylating downstream targets such as AS160, which facilitates membrane translocation of GLUTs, further increasing glucose uptake. AKT also inhibits gluconeogenesis and enhances glycogen synthesis by phosphorylating glycogen synthase kinase 3 (GSK3), linking growth factor signaling to energy storage pathways 136, 137.

The *PI3K/AKT/mTOR* pathway also plays a pivotal role in lipid metabolism. AKT activates SREBP1c, a master regulator of lipogenesis, leading to the upregulation of lipogenic enzymes such as FASN and ACC. mTORC1 indirectly controls SREBP1 nuclear translocation through the activation of *S6K1* and *CREB*, reinforcing fatty acid synthesis 138. In parallel, mTORC1 inhibits lipolysis, promoting energy storage to sustain tumor growth. mTORC2 also modulates lipid metabolism by phosphorylating AKT, which in turn suppresses *Forkhead box C2 (FoxC2)*, a transcription factor that regulates lipid homeostasis. Additionally, mTORC1 promotes fat synthesis through downstream regulation of PPARγ and Lipin1, further enhancing lipid availability to fuel tumor progression 139. Dysregulated lipid metabolism not only provides energy but also generates membrane components and signaling lipids, supporting tumor growth and metastasis.

The mTORC1 complex integrates amino acid availability to regulate protein synthesis and cellular growth. Among amino acids, leucine is a critical activator of mTORC1. Leucine binds to Sestrin2, disrupting its inhibitory interaction with the GATOR2 complex, leading to mTORC1 activation. This activation drives protein synthesis via phosphorylation of downstream effectors such as S6 kinase 1 (S6K1) and eIF4E-binding protein 1 (4EBP1), thereby promoting ribosomal biogenesis and translation of growth-related mRNAs 140, 141.

Conversely, under nutrient-deprived conditions, mTORC1 inhibition triggers autophagy to recycle cellular components and maintain energy balance. Autophagy provides an alternative energy source during metabolic stress, highlighting the dual role of mTORC1 in balancing anabolism and catabolism. 142, 143.

In addition to protein synthesis, the *PI3K/AKT/mTOR* pathway regulates glutamine metabolism, the TCA cycle, and the PPP. mTORC1 enhances glutaminolysis to fuel the TCA cycle, providing essential intermediates for biosynthetic pathways. Furthermore, activation of the PPP generates NADPH and ribose-5-phosphate, which are critical for redox homeostasis and nucleotide synthesis.

The *PI3K/AKT/mTOR* signaling pathway plays a central role in orchestrating cancer cell metabolism, integrating signals to regulate glycolysis, lipid synthesis, protein synthesis, and amino acid metabolism. Through its downstream effectors, mTORC1 and mTORC2, this pathway ensures metabolic flexibility, enabling cancer cells to thrive under diverse conditions. Targeting the *PI3K/AKT/mTOR* axis offers significant therapeutic potential, as evidenced by the clinical success of drugs such as Alpelisib and Everolimus. Future strategies should focus on overcoming therapy resistance through combination treatments, paving the way for more personalized and effective cancer therapies 144.

#### HIF-α, MYC, and metabolic adaptation

TME is characterized by hypoxia and acidity, largely driven by the rapid proliferation of tumor cells and the Warburg effect. HIF-1α is a master regulator that enables cancer cells to adapt to hypoxic conditions by orchestrating transcriptional programs. Under normoxic conditions, HIF-1α is hydroxylated by proline hydroxylase (PHD), facilitating its interaction with von Hippel-Lindau protein (pVHL), which tags HIF-1α for proteasomal degradation. In hypoxia, PHD activity is suppressed, stabilizing HIF-1α, which then dimerizes with HIF-1β to bind hypoxia response elements (HRE) in target gene promoters, activating pathways involved in glycolysis, angiogenesis, and survival 145, 146.

HIF-1α plays a pivotal role in tumor metabolism by promoting glycolysis through the upregulation of *GLUT1, HK2, and LDHA*. Furthermore, it enhances angiogenesis by stimulating VEGF expression, promoting nutrient supply to hypoxic regions of tumors. Importantly, HIF-1α also affects immune cell function within the TME. HIF-1α not only helps tumor cells adapt to the hypoxic environment, but also helps T cells adapt to the hypoxic environment, enhancing the killing effect of T cells on tumors, and the absence of HIF-1α impairs the cytotoxicity of T cells. Targeting HIF-1α has been shown to suppress PD-L1 expression on tumor and myeloid cells, enhancing the efficacy of ICIs like anti-CTLA-4 antibodies 147, 148.

MYC, encoded by the proto-oncogene *MYC*, is another critical transcription factor that cooperates with HIF-1α to drive metabolic adaptation. MYC regulates genes involved in glucose uptake (*GLUT1*) and glycolysis (*HK2, PFK1*), enhancing the glycolytic capacity of tumor cells. Under hypoxic conditions, HIF-1α and MYC act synergistically to promote the expression of PKM2, a key enzyme that regulates the balance of glycolytic intermediates between ATP production and biosynthetic pathways, such as the PPP and serine biosynthesis 149, 150.

In addition to affecting glycolysis, MYC also reprograms lipid metabolism by interacting with SREBP1, driving fatty acid and cholesterol synthesis. MYC upregulates 3-hydroxy-3-methyl-glutaryl coenzyme A reductase (HMGCR), the rate-limiting enzyme in cholesterol biosynthesis, further enhancing tumor cell proliferation and survival 151. Interestingly, HIF-1α and MYC exhibit a dynamic interplay. While MYC can upregulate HIF-1α under certain conditions, HIF-1α can also inhibit MYC activity by activating MXI-1, a MYC antagonist, to prevent excessive cell growth under hypoxia 152 153.

HIF-1α and MYC are two core transcription factors for tumors to adapt to the hypoxic microenvironment. They jointly drive tumor progression and survival through the coordinated regulation of glycolysis, lipid metabolism, and angiogenesis.

#### Cancer stem cell metabolic characteristics and their regulation

CSCs constitute a subpopulation of cancer cells endowed with self-renewal capacity, multipotency, and resistance to therapy. Metabolic reprogramming is critical for the maintenance of CSCs, allowing them to generate sufficient energy and biosynthetic precursors for survival and proliferation. Unlike differentiated tumor cells, CSCs exhibit remarkable metabolic plasticity, shifting between glycolysis and oxidative phosphorylation (OXPHOS) based on microenvironmental conditions.

Lipid metabolism plays a particularly vital role in CSC biology. CSCs display elevated lipid synthesis and storage, contributing to chemoresistance, invasion, and metastasis. Reprogramming of lipid metabolism enhances CSC membrane fluidity and mitochondrial function, enabling adaptation to metabolic stress 154-156. Autophagy, a key regulator of lipid turnover in CSCs, further supports survival by providing energy and substrates during nutrient deprivation. While autophagy can suppress tumorigenesis, in CSCs it often enhances stemness, survival, and therapy resistance 157, 158.

The signaling pathways that regulate CSCs include the *Wnt/β-catenin, PI3K/AKT/mTOR*, *Notch*, *Hedgehog*, and *JAK/STAT3* pathways. In breast cancer and pancreatic cancer, Notch signaling is activated and contributes to the maintenance of stemness in CSCs. Hif-1α-activated* Notch* signaling can promote CSCs-associated tumor metastasis in lung, ovarian, and breast cancer. Moreover, it has been shown that many genes expressed by CSCs can, in turn, activate *Notch* signaling 159, 160.

The* WNT/β-catenin* signaling pathway involves the binding of WNT ligands to Frizzled and low-density lipoprotein receptor-related protein (LRP) receptors, thereby disinhibiting β-catenin. Meanwhile, overexpression of the *WNT/β-catenin* signaling pathway can maintain CSC stemness, leading to resistance to related therapies 161.

The *JAK/STAT3* signaling pathway is involved in many cellular physiological processes. It has been shown that *JAK2/STAT3* signaling upregulates cyclin D2 and stemness-related transcription factors, maintains CSC stemness, and promotes tumor metastasis through mesenchymal transition (EMT). Inhibition of the *JAK2/STAT3* signaling pathway can impair the stemness of CSCs 162-164.

Moreover, many other signaling pathways do not work independently but interweave and regulate each other through complex networks to jointly influence the properties and behavior of CSCs. In summary, HIF-1α and MYC are key drivers of metabolic reprogramming, enabling cancer cells to adapt to hypoxia and sustain growth through glycolysis, lipid synthesis, and biosynthesis. In parallel, CSCs exhibit metabolic flexibility, relying on lipid metabolism, autophagy, and key signaling pathways to maintain stemness and therapy resistance. Targeting these interconnected pathways provides a promising therapeutic strategy for disrupting tumor progression and overcoming treatment resistance.

### 3.2 Metabolic reprogramming and tumor microenvironment

#### Role of immune-cell metabolism in urologic cancers

The metabolic environment (such as pH, hypoxic environment, etc.) in the TME and the cytokines and chemokines secreted by the tumor jointly affect the function of immune cells in the microenvironment, resulting in immune cells eventually playing anti-tumor or pro-tumor roles. Neutrophils recruited in the TME can exert anti-tumor effects by promoting the expression of genes such as* CXCL1*, *CXCL2*, and *CXCL5* 165.

TAMs are among the most abundant immune cells in the TME and exhibit functional plasticity with two major polarization states: M1-like TAMs, which are pro-inflammatory and anti-tumorigenic, and M2-like TAMs, which are immunosuppressive and pro-tumorigenic 166. M2-like TAMs promote tumor progression by secreting pro-angiogenic factors such as VEGF and PDGF, activating vascular endothelial growth factor receptor 2 (VEGFR-2) on endothelial cells to drive angiogenesis. Additionally, M2 TAMs secrete immunosuppressive cytokines such as TGF-β and TNF-α, which suppress immune surveillance and facilitate tumor immune evasion 167, 168.

Metabolic competition within the TME also influences TAM function. For example, TAMs preferentially utilize glucose, promoting O-GlcNAcylation of Cathepsin B, enhancing its maturation and contributing to metastasis and chemotherapy resistance in mouse models 169. In contrast, M1-like TAMs rely on glycolysis to produce cytotoxic mediators, underscoring the potential of metabolic reprogramming to shift TAMs toward a tumor-suppressive phenotype.

Tumor-associated neutrophils (TANs), like TAMs, display functional polarization into two phenotypes: N1-like TANs, which are anti-tumorigenic, and N2-like TANs, which are pro-tumorigenic. N1-like TANs can directly kill tumor cells and inhibit their proliferation by secreting large amounts of ROS and hypochlorous acid 170. Meanwhile, N1-like TANs exhibit anti-tumor activity by enhancing T cell responses and producing pro-inflammatory cytokines.

However, interestingly, low concentrations of ROS can promote tumor growth and related signaling, while high concentrations of ROS have damaging effects on tumor cells, leading to genetic toxicity and pro-apoptotic effects 171. N2-like TANs promote tumor progression through multiple mechanisms, including the secretion of IL-1β, matrix metalloproteinases (MMPs), and neutrophil elastase, which enhance tumor cell migration, invasion, and metastasis by remodeling the ECM and facilitating extravasation of cancer cells into pre-metastatic niches 172. The polarization and metabolic rewiring of TANs are regulated by environmental factors in the TME, such as hypoxia and nutrient availability, providing a potential therapeutic opportunity to reprogram TANs toward an anti-tumor phenotype 173.

Other immune cells, such as eosinophils and mast cells, further contribute to the metabolic reprogramming of the TME. Eosinophils exert anti-tumor activity through cytotoxic granules but can promote immunosuppression by secreting IL-4 and IL-13, favoring M2 macrophage polarization. Similarly, mast cells secrete pro-angiogenic mediators like VEGF and histamine, promoting tumor progression, while under certain conditions, they enhance T cell recruitment and anti-tumor immunity. This duality highlights the complexity of immune cell functions in the metabolically reprogrammed TME.

The interplay between immune-cell metabolism and the TME provides promising therapeutic opportunities in urologic cancers. Strategies aimed at reprogramming immune cell metabolism—such as targeting fatty acid oxidation (FAO) in TAMs, inhibiting neutrophil elastase to reduce TAN pro-tumor effects, and modulating cytokine production—can shift the immune balance toward anti-tumor activity. Combining metabolic therapies with ICIs has shown potential to synergistically enhance immune responses, representing a promising approach to improve treatment outcomes in urologic malignancies 174-176.

#### Metabolic effects of tumor-stroma interactions

Tumor stroma consists of ECM components and a variety of cell populations. It plays a crucial role in the occurrence, development, metastasis, and drug resistance of tumors. Stromal cells include CAFs, cancer-associated adipocytes, and cancer-associated endothelial cells, among which CAFs are the most important cells. CAFs can construct ECM (which can lead to matrix sclerosis) and secrete many cytokines and chemokines (such as CCL2, CCL5, and CXCL5) 177, 178.

Fibroblasts and mesenchymal stromal cells in the tumor stroma secrete cytokines such as HGF and FGF to promote angiogenesis, whereas stromal-related factors such as ISF-1 and IGF-2 promote tumor cell infiltration 179. Among them, the competition between tumor cells and T cells for glucose can lead to the hyporeactivity of T cells, leading to immune suppression and thereby promoting tumor progression 180, 181. In addition, glycolysis and mitochondrial activity in CAFs are increased due to increased stiffness, which promotes lactate secretion, and TCA cycle intermediates are increased. These findings suggest that matrix hardening promotes glucose metabolic reprogramming 182. However, there was no significant change in mitochondrial activity in tumor cells.

For amino acid metabolism, studies have shown that tumor cells secrete glutamate (Glu) and absorb aspartic acid (Asp), whereas CAFs secrete Asp and absorb Glu. Both tumor cells and CAFs have increased glutamine (Gln) consumption in sclerotic stroma, and the expression of Gln metabolism-related genes is up-regulated. The Gln-dependent exchange of Asp and Glu between CAFs and tumor cells promotes tumor activity. CAF-derived ASPs maintain cancer cell proliferation, while cancer cell-derived GLS balances the REDOX state of CAF to promote ECM remodeling 182. It has also been shown that autophagy in CAFs can produce alanine, which can be used by pancreatic ductal adenocarcinoma (PDAC) cells to promote the TCA cycle 183.

The presence of stromal cells reduces the efficacy of drug therapy by increasing interstitial hydraulic pressure and regulating the ability of chemical drugs to cross capillaries. Moreover, stromal cells can directly reduce the sensitivity of tumor cells to chemotherapeutic drugs and tyrosine kinase inhibitors 184.

In summary, the stiffness of the matrix in TME can regulate the function of tumor cells. On the one hand, sclerosing ECM inhibits the infiltration and function of immune cells, promoting drug resistance in tumor cells. On the other hand, sclerosing ECM promotes glucose metabolism reprogramming or amino acid exchange, as well as the interaction between stromal cells and tumors, jointly promoting tumor progression.

## 4. Metabolic reprogramming as a therapeutic target

### 4.1 Progress in metabolic targeted therapy

#### Metabolic inhibitors in bladder cancer

The drug treatment of bladder cancer mainly includes the following categories: chemotherapy drugs, targeted therapy drugs, ICIs, metabolic inhibitors, and ADC drugs. Chemotherapy regimens are generally divided into the following: 1. Gemcitabine combined with cisplatin (GC regimen); 2. ddMVAC (dose-dense methotrexate, vinblastine, doxorubicin, and cisplatin) combined with growth factors; 3. CMV regimen (cisplatin, methotrexate, and vinblastine).

Metabolic inhibitors are currently a hot research field. For glucose metabolism, 2DG, an HK2 inhibitor, has been shown to reduce bladder cancer cell viability, proliferation, migration, and invasion *in vitro* and *in vivo* preclinical UBC models 58. It has also been shown that oridonin forms a covalent bond with Cys-813 near the glucose-binding domain of HK1, thereby inhibiting its enzymatic activity. This enhances the expression of lactic acid, thereby alleviating the immunosuppressive effect of lactic acid on CD8+ cells. Oridonin combined with a PD-L1 inhibitor can also enhance the killing effect of CD8+T cells on bladder cancer. These results suggest that targeting HK1 and HK2 may be potential targets for the treatment of bladder cancer 185. Similarly, inhibition of PFK-1 inhibited the proliferation of bladder cancer cell line T24. However, no effective PFK-1 inhibitor has been used in clinical patients due to its safety 186.

For amino acid metabolism, studies have found that chemotherapy can upregulate the expression of tryptophan metabolism enzyme IDO1 and tryptophan transporter SLC7A5, which enhances the tumor uptake of tryptophan and its metabolism to the downstream product kynurenine (Kyn). Kyn inhibits STING-dependent type I interferon production by enhancing STING protein degradation through activation of the "AhR-CUL4B-RBX1" E3 ubiquitin complex. Dietary tryptophan restriction, blockade of the key rate-limiting enzyme IDO1 of tryptophan metabolism, or inhibition of cellular tryptophan import also contribute to the inhibition of tumor progression, suggesting that tryptophan metabolism plays an important role in chemotherapy resistance in bladder cancer, which provides a new perspective for the development of therapeutic strategies for bladder cancer targeting the tryptophan metabolism pathway 187.

Lipid metabolism reprogramming is one of the important reasons for the progression and metastasis of bladder cancer; thus, regulating lipid metabolism is highly important for the treatment of bladder cancer. For lipid metabolism, FASN is highly expressed in bladder cancer, and FASN has a high targeting ability in bladder cancer. The use of FASN inhibitors for the treatment of bladder cancer is expected to have high efficacy and fewer toxic side effects. The efficacy and safety of TVB-2640 in anti-tumor have been clinically investigated in phase I clinical trials 96, and FASN inhibitors were developed for bladder cancer. FASN inhibitors affect tumor lipid metabolism, suggesting their promise in combination with other agents such as ICIs. Current drugs include cerulenin, orlistat, TVB-2640, and so on. TVB-2640 combined with bevacizumab has shown positive results in a phase II clinical trial of recurrent glioblastoma in patients with first recurrence of high-grade astrocytoma (clinical trial registration number: NCT03032484). FASN may be a potential therapeutic target for bladder cancer, and inhibition of FASN expression is expected to become a new method for the treatment of bladder cancer. In addition, validated through clinical trials, Erdafitinib (Balversa) has been approved by the FDA for locally advanced or metastatic urothelial carcinoma (UC) carrying susceptible FGFR3 or FGFR2 gene alterations, making it the first FGFR kinase inhibitor in adult patients. This finding highlights the potential and application value of erdafitinib in the treatment of bladder cancer instead of chemotherapy 188.

The treatment of bladder cancer is developing from traditional chemotherapy to more precise targeted and immunotherapy. Inhibitors targeting metabolic reprogramming (sugar, amino acid, lipid metabolism) are new strategies with great potential. They can not only directly kill tumor cells, but also regulate the tumor microenvironment, overcome drug resistance, and synergize with existing immunotherapies.

#### The potential of targeting lipid metabolism in renal cell carcinoma

Renal cell carcinoma is considered a metabolic disease, and fatty acid metabolism is important in the progression of ccRCC. Targeting fatty acid metabolism may be a potential way to reverse drug resistance and improve the prognosis of ccRCC. GPR1 and CMKLR1 of G protein-coupled receptors (GPCRs) are involved in the regulation of lipid metabolism in clear cell renal cell carcinoma (ccRCC). They limit lipolysis by inhibiting adipose triglyceride lipase (ATGL), and CMKLR1 regulates lipid uptake. Inhibition of CMKLR1 inhibited lipid formation and induced cell death, including apoptosis, ferroptosis, and autophagy. Targeted inhibition of CMKLR1 via 2- (α-naphthyl) ethyltrimethylammonium iodide (α-NETA) significantly inhibited ccRCC growth 189.

On the other hand, it has been suggested that PHF8 is an important regulator of lipid deposition in ccRCC. Phf8 promotes lipid deposition by transcriptionally upregulating glutamic acid ammonia ligase (GLUL). GLUL inhibitor L-methionine sulfoxide (MSO), combined with everolimus, can effectively inhibit lipid deposition and tumor growth in renal cell carcinoma, providing a new treatment strategy for renal clear cell carcinoma 111. Stearoyl-CoA desaturase 1 (SCD1) can desaturate saturated fatty acids (SFA) to monounsaturated fatty acids (MUFA), thereby promoting ccRCC progression 190. Inhibition of SCD1 can limit the growth of tumor cells, and the combination of SCD1 inhibitors with mTOR inhibitors may have an inhibitory effect on lipid metabolism in renal cancer, which may lead to a new therapeutic approach 191.

The key regulatory factor of fatty acid synthesis metabolism, malonyl CoA decarboxylase (MLYCD), is significantly downregulated in renal clear cell carcinoma, and low expression is associated with poor prognosis in patients. Restoring MLYCD expression in renal clear cell carcinoma cells reduces intracellular malonyl CoA content, inhibits de novo synthesis of fatty acids, and promotes fatty acid transfer to mitochondria for oxidation. Overexpression of MLYCD can block lipid droplet accumulation in cancer cells, disrupt endoplasmic reticulum and mitochondrial homeostasis, increase reactive oxygen species levels, and induce ferroptosis. In addition, overexpression of MLYCD reduced tumor growth and reversed resistance to sunitinib *in vitro* and *in vivo* 192.

The above studies all suggest that targeted therapy targeting lipid metabolism reprogramming has the potential to become a new therapy for renal clear cell carcinoma, which requires further clinical research exploration.

#### Strategies for combining metabolic interventions with prostate cancer treatment

Nonsurgical treatment of prostate cancer is typically ADT, which includes the use of AR inhibitors (e.g., bicalutamide, apalutamide) and androgen synthesis inhibitors (e.g., abiraterone). GnRH agonists induce down-regulation and desensitization of GnRHR by inducing sustained stimulation of the pituitary gland, resulting in decreased luteinizing hormone (LH) release and suppression of testosterone production to castrate levels. This class of agents (e.g., goserelin and leuprolide) is now widely used in patients with advanced prostate cancer to control disease progression 193, 194.

Moreover, prostate tumors are highly dependent on lipids for energy, growth, and survival. It has been reported that ACSM1 and ACSM3 are directly regulated by AR in prostate cancer, and they promote FAO to fuel cancer cells. These two enzymes enhance proliferation and protect PCa cells from death *in vitro*. Targeting ACSM3 resulted in reduced tumor growth, revealing a link between AR and lipid metabolism, and future development of drugs targeting ACSM1 and ACSM3 is expected to explore new therapies in combination with traditional ADT therapy 116.

In addition, ATGL is an enzyme that controls lipid droplet homeostasis, and its expression is associated with poor overall survival in patients with CRPC. The data suggest that ATGL can be used as a therapeutic target for CRPC, and the main problem is to target the potential effect of ATGL on the heart, which also provides a new perspective for the treatment of prostate cancer metabolism 195.

For amino acid metabolism, the protein expression level of branched-chain amino acid transaminase 2 (BCAT2) is significantly increased in prostate cancer cells, which allows cancer cells to more efficiently use branched-chain amino acids (BCAAs) as energy sources and raw materials for biosynthesis, thereby promoting the rapid growth of cancer cells. This also provides ideas for the subsequent development of targeted drugs for the treatment of prostate cancer 196.

In summary, the reprogramming of lipid metabolism and amino acid metabolism in prostate cancer has been studied, demonstrating the potential of metabolic intervention in the treatment of prostate cancer. By targeting specific metabolic pathways and enzymes, more effective treatment options for prostate cancer patients can be provided in the future.

### 4.2 Combination of metabolic targets and immunotherapy

#### Synergy of metabolic regulation and immune checkpoint inhibitors

TME is a metabolically hostile niche where cancer cells exploit immune checkpoints to evade immune surveillance. ICIs, such as anti-PD-1/PD-L1 and anti-CTLA-4 therapies, have revolutionized cancer treatment by reactivating T cell-mediated immunity. However, a substantial proportion of patients fail to derive clinical benefits from ICIs due to factors such as metabolic competition in the TME, which suppresses immune cell function.

Tumor cells consume excessive glucose, limiting nutrient availability for T cells and impairing their glycolytic activity, a critical process for effective T cell activation and function. PD-1 signaling plays a pivotal role in T cell metabolic reprogramming. It inhibits glucose uptake and glycolysis while promoting FAO via the* AMPK-CPT1A* axis. This metabolic shift reduces T cell effector function and promotes exhaustion 197. Notably, PD-L1 expressed on tumor cells directly enhances tumor glycolysis by activating the *PI3K/AKT/mTOR* pathway, creating a metabolic advantage for tumor survival and further suppressing T cell activity 198.

Therefore, targeting PD-1/PD-L1 not only restores T cell glycolysis and effector function but also reduces tumor glucose consumption, creating a synergistic anti-tumor effect. These insights highlight the therapeutic potential of combining ICIs with glucose metabolism inhibitors to reprogram the TME and improve immunotherapy outcomes.

In addition to glucose metabolism, amino acid metabolism, particularly arginine (Arg), plays a critical role in immune suppression within the TME. Arginine depletion by myeloid-derived suppressor cells (MDSCs) leads to impaired T cell proliferation and function. Recent studies have shown that CB-1158, an orally active arginase inhibitor, restores arginine availability, promoting the infiltration of CD8+ T cells and NK cells into the tumor. CB-1158 not only reduces myeloid cell-mediated immune evasion but also enhances tumor growth inhibition when combined with PD-L1 blockade or chemotherapy (e.g., gemcitabine) in preclinical models (186). This evidence underscores the importance of targeting arginine metabolism as a strategy to synergize with ICIs, enhancing immune activation and tumor clearance 199.

Lipid metabolism is another critical axis influencing immune function in the TME. Tumor-associated dendritic cells (TADCs), under lipid overload conditions, exhibit impaired antigen presentation, contributing to immune evasion 200-202. An innovative approach using multi-layer lipid reprogramming nanoparticles (TS-PP@FU) has been developed to specifically deliver lipid metabolism inhibitors to TADCs. These nanoparticles synergistically inhibit exogenous lipid uptake, endogenous lipid synthesis, and lipogenic gene transcription in TADCs, thereby restoring their anti-tumor immune function. Furthermore, this strategy enhances the efficacy of ICIs, such as anti-PD-1 monoclonal antibodies, and overcomes resistance to immune checkpoint blockade 203. The integration of lipid metabolism-targeting strategies with ICIs represents a promising approach to improve immunotherapy outcomes. By modulating lipid reprogramming in immune cells, this combination therapy reshapes the immune landscape of the TME, restoring dendritic cell function, improving T cell activation, and amplifying the anti-tumor response.

The combination of metabolic regulation and ICIs represents a powerful strategy for enhancing anti-tumor immunity in cancer therapy. Targeting glucose metabolism restores T cell function and reduces tumor metabolic dominance, while inhibiting arginine metabolism alleviates immunosuppression mediated by myeloid cells. Additionally, interventions in lipid metabolism improve dendritic cell function and overcome resistance to ICIs. These synergistic approaches provide new avenues for reprogramming the TME, overcoming immune evasion, and enhancing the efficacy of immunotherapy in urologic cancers. Future research focusing on the identification of precise metabolic targets and their integration with ICIs will accelerate the development of personalized combination therapies for cancer patients.

#### Impact of metabolic reprogramming on immune escape

Tumor cells change their energy metabolism to adapt to the hypoxic and nutrient-deficient microenvironment through metabolic reprogramming. The main features of metabolic reprogramming include abnormal glucose metabolism, amino acid metabolism, and lipid metabolism, which are the key factors leading to TME immunosuppression and tumor immune escape.

Glucose is an important source of cellular energy, and tumor cells tend to produce energy through the glycolytic pathway, which is called the Warburg effect 17. The high lactate content and the concomitant acidified TME in tumors will inhibit the function of immune cells, cancel the immune surveillance of cancer, and eventually lead to immune escape. CD8+T cells are key mediators of anti-tumor immunity, and after continuous stimulation of the T cell receptor (TCR) in the TME, they lead to T cell exhaustion and eventually tumor immune escape 204. However, the latest research has found that CD8+T cells are not immune. Exhausted T cells highly express the solute carrier (SLC) protein MCT11, promoting their uptake of lactate, and blockade of MCT11 restores T cell function 205. And HK2 not only participates in glycolysis, but also activates the NF-κB pathway, promotes PD-L1 expression, and leads to tumor immune escape. The combination of HK2 inhibitors and PD-1 antibodies can significantly enhance the activity of CD8+T cells and improve therapeutic efficacy 206.

In amino acid metabolism, Gln deficiency inhibits T cell proliferation and cytokine production, while supplementation with Gln precursors does not restore the phenotype, suggesting that T cells are heavily dependent on extracellular Gln uptake 207. Tumor cells exhibit strong uptake of Gln, leading to a decline in the function of tumor-infiltrating lymphocytes (TILs) and contributing to tumor immune escape 53. In addition to Gln, Arg and Asn are also depleted in TME, and Arg deficiency leads to a bias of T cell metabolism away from OXPHOS toward glycolysis, attenuating T-cell antitumor activity 50. Similar to the use of Gln, tumor cells use Asn to promote their own proliferation, while depletion of Asn impairs CD8+T cell activation 208. Furthermore, many amino acid deficiencies jointly lead to tumor proliferation and immune escape through various pathways 209.

Lipid metabolism reprogramming is not limited to tumor cells, but is closely related to the function of immune cell infiltration into TME, and lipid accumulation in dendritic cells induces endoplasmic reticulum stress, which reduces antigen presentation 210. Lipids are closely related to various immune cells and their phenotypic transformation in TME. Lipid metabolism reprogramming in the TME increases lipid absorption and oxidation to increase the efficiency of energy metabolism in tumor cells while limiting the nutritional source of CD8+ T cells and impairing their function 211. For T cells, lipid accumulation is associated with increased CD36 expression on CD8 TILs, which promotes OxLDL uptake. Promotes lipid peroxidation as well as p38 activation. It causes dysfunction of T cells 212. In addition, endoplasmic reticulum stress induced by cholesterol accumulation leads to T-cell exhaustion, which promotes tumor immune escape 213. In contrast, inhibition of lipid metabolism can restore T cell antitumor responses. Recent studies have found that blocking the sphingolipid production pathway in cancer cells, especially through the regulation of interferon-γ (IFN-γ) signaling pathway, can effectively enhance the anti-tumor proliferation efficacy of natural killer cells and CD8+T cells 214.

In summary, the three major metabolic reprogramming processes of tumor cells, on the one hand, can take nutrients from TME to maintain their proliferation and invasion, on the other hand, they can promote the downregulation or depletion of TILs, thereby promoting immune escape. Studies have also shown that the regulation of metabolism can restore the anti-tumor response to a certain extent, which makes us look forward to the combination of metabolic inhibitors with other targeted drugs in the future, so that patients can achieve better efficacy.

## 5. Future prospects

### Prospects for exploration and application of novel metabolic markers

Metabolic reprogramming is one of the hallmark features of tumor cells. In urinary system tumors (such as ccRCC, BCa, etc.), tumor cells meet the energy and material requirements of rapid proliferation by changing their metabolic mode (such as glycolysis, amino acid metabolism, lipid metabolism, etc.), and adapt to the tumor microenvironment. These metabolic changes are often driven by specific gene mutations or signaling pathway abnormalities, and leave specific clues, that is, potential biomarkers.

Current research has revealed reprogramming of multiple metabolic pathways in urinary tract tumors and identified some potential biomarkers from them.

For example, in lipid metabolism, studies have shown that MLYCD, FASN, ACSM, and ACSL play important roles in the progression of urinary tract tumors. Some of these molecules have also been explored in clinical drug research, such as TVB-2640 and other drugs.

Among the biomarkers related to amino acid metabolism, histone deacetylase 7 (HDAC7) can suppress the expression of branched chain amino acid (BCAA) catabolizing enzymes such as BCAT2 and BCKDHA through epigenetic means, leading to BCAA accumulation, activating the NOTCH signaling pathway and upregulating the SNAIL1 transcription factor, promoting epithelial mesenchymal transition (EMT) and renal tumor metastasis 215. In bladder cancer, the acquired mutation of isocitrate dehydrogenase 2 (IDH2) can induce reductive glutamine metabolism, stabilize the expression of HIF-1α, thus stimulate aerobic glycolysis and pentose phosphate pathway (PPP), and promote gemcitabine resistance 216. Regarding other metabolic pathways, studies have also shown that high expression of peroxisome proliferator-activated receptor gamma coactivator 1 alpha (PGC-1 alpha) is associated with enhanced mitochondrial biosynthesis and oxidative phosphorylation, which can promote cancer metastasis. Interventions targeting PGC-1α may inhibit metastasis 217.

Among the phosphoinositide (PI) metabolism-related markers, the study found that the phosphoinositide metabolism score (PIMS) was related to the prognosis of urinary system cancer (kidney cancer, bladder cancer, prostate cancer), and the prognosis of patients with low PIMS was poor. PNPLA7 has been identified as a core prognostic gene in PI metabolism, and its expression loss is closely related to tumor progression 218.

For bladder cancer, urinary metabolic markers are of great value for the diagnosis and prognosis of bladder cancer. Studies have shown that metabolites in the urine can reflect the presence of a tumor or the body's response to the tumor, such as reduced hippuric acid and citrate levels 219. Hematuria is a common symptom of urological tumors, for which studies have found that 4-ethoxymethyl phenol, prostaglandin F2b, etc., help to distinguish BC from RCC 220. However, no urinary metabolites have been found to distinguish BC from PCa. The current research progress has not found that urine metabolites are helpful for the diagnosis and prognosis of urological tumors.

In summary, the research progress of metabolic markers in urinary system tumors provides new strategies and methods for early diagnosis, prognosis evaluation, and treatment of tumors, and shows broad application prospects. In the future, more potential metabolic markers are expected to be found for the detection and clinical treatment of urologic tumors. Most of the evidence summarized in the previous article is based on preclinical or retrospective studies; Various drugs targeting metabolic reprogramming still need to be prospectively validated in clinical trials to confirm the translational potential of metabolic targets.

### Clinical translation and challenges of metabolic reprogramming targets

The development of drugs targeting the metabolic reprogramming of tumor cells is one of the feasible methods for the treatment of tumors. It can be divided into three aspects: targeting tumor cells, targeting TME, and regulating systemic metabolism. For the characteristics of active proliferation of tumor cells, interfering with nucleotide metabolism is a feasible way. Synthetic lethality refers to the phenomenon that two nonlethal genes are inhibited at the same time, leading to cell death. PARP inhibitors (PARPi) are effective in inhibiting tumor proliferation in BRCA1/2 mutated tumors. Due to the homologous recombination repair defect (HRD) caused by the mutation of the tumor BRCA1/2 gene, the DNA cannot be repaired by the base excision repair (BER) pathway and homologous recombination repair (HRR) pathway after the use of PARPi, which eventually leads to cell death. In clinical trials, patients with breast, ovarian, pancreatic, and prostate cancers, including those with BRCA germline mutations, have benefited substantially from the use of PARPi 221. A phase III clinical study (NCT03732820) of olaparib combined with abiraterone in the first-line treatment of mCRPC showed that the combination treatment of mCRPC patients without HRR mutation screening could significantly prolong rPFS by 8.2 months (24.8 vs 16.6 months) and reduce the risk of disease progression or death by 34% In addition, the quality of life of patients did not significantly decline. This finding suggests that synthetic lethal strategies have important potential in combination therapy for treating urologic tumors 222. But as mentioned earlier, there are still many targets that we can explore, such as FASN inhibitors, HK1, and HK2 inhibitors, etc.

At present, there are many ongoing clinical trials and biomarker validation related to urinary system cancer. For prostate cancer, the FASN inhibitor tvb-2640 is the most representative. At present, tvb-2640 is in phase I clinical trial (NCT05743621), to explore the prognostic impact of enzalutamide combined with tvb-2640 on prostate cancer. In addition, pasritamig (JNJ - 78278343) is currently in phase I clinical trial (NCT04898634) and plans to start phase II clinical trial. This is a bispecific antibody that can simultaneously target kallikrein 2 (klk2) on the surface of tumor cells and CD3 on the surface of T cells. It acts like a "bridge" to pull T cells to tumor cells and activate the killing effect of T cells on tumors. According to the data published in phase I, 42.4% of patients achieved PSA50 response, with a median RPFS of 7.9 months, and the safety was good, without high-grade cytokine release syndrome. Meanwhile, the phase IB study of pasritamig combined with another psma-cd28 bispecific antibody JNJ-87189401 has been started (NCT06095089).

For bladder cancer, a phase II clinical study (NCT04813107) evaluated oral apl-1202 (a methionine aminopeptidase type II inhibitor) combined with tirelizumab (PD-1 inhibitor) compared with tirelizumab alone. The results showed that the pathological complete response rate (pCR) of the combined treatment group and the single agent group were 41% and 20%, respectively. Notably, in the subgroup with low expression of PD-L1, the combination regimen showed a stronger positive efficacy signal. It is worth noting that its safety as neoadjuvant therapy for MIBC patients is generally controllable, although the incidence of treatment-related adverse events (TRAEs) at any level in the combination group (59%) is higher than that in the single agent group (44%). However, 94% of TRAEs were grade 1 or 2 events, that is, the degree was mild, and patients were generally able to tolerate them.

For renal cancer, there are few clinical studies on targeted drugs for metabolic reprogramming, and there are many preclinical studies at present. Targeting the FABP1-PLG-PLAT axis inhibits fatty acid metabolic reprogramming and tumor angiogenesis by inhibiting fatty acid binding protein 1 (FABP1) and its downstream plasminogen (PLG) - tissue plasminogen activator (PLAT) signaling pathway. Targeted inhibition of monoglyceride lipase (MGLL) affects lipid metabolism and tumor microenvironment.

But there are many challenges faced in developing drugs. First, metabolic enzymes may have different functions in different tissues. Targeting metabolic enzymes or metabolic pathways can inevitably damage normal cells. For example, classical LDH has five isoenzyme forms, which play different functions in different tissues. Targeting ATGL as described above may be a feasible treatment for CRPC, but its potential effects on the heart may lead to serious drug side effects. This means that the study of metabolic pathways in different pathological models cannot be generalized, which brings great difficulties to the development of drugs.

However, there are also successful cases, such as the up-regulation of FASN expression in lung cancer, which is associated with poor prognosis, and inhibitors targeting FASN, such as orlistat, can inhibit tumor growth by preventing EGFR palmitoylation and enhancing its ubiquitination 223.

Another challenge is the metabolic fitness and heterogeneity of tumors, which can shift tumors to utilize other nutrients for function. Therefore, drugs targeting the metabolic profile of primary tumors may not be effective in metastatic tumors and may lead to drug resistance. In addition, differences in metabolism within and between tumors may also reduce drug efficacy, and there may also be differences in metabolism between different patients with the same tumor. All of these aspects make drug development difficult. In summary, metabolic reprogramming targeting therapy has shown great potential in clinical translation, but it also faces many challenges, including an in-depth understanding of the specific mechanism of metabolic reprogramming, the development of highly specific small-molecule targeted drugs, and the optimization of clinical treatment strategies based on tumor metabolism dependence.

## 6. Summary

### The critical role of metabolic reprogramming in urologic cancers

For most tumors, the Warburg effect of tumor cells promotes an acidic TME environment, which leads to downregulation of immune cell function and tumor immune escape. For T cells, abnormal amino acid metabolism and lipid metabolism can lead to the downregulation of T cell function or the exhaustion of T cells. In bladder cancer, the reprogramming of glucose metabolism rapidly provides ATP, glutamine, serine, arginine, fatty acids, and lipids to the tumor to promote its proliferation. Targeted regulation of certain processes of glucose metabolism reprogramming in bladder cancer cells (such as targeting HK2) can inhibit the proliferation of bladder cancer cells, and abnormal glucose metabolism may contribute to drug resistance to chemotherapy drugs. Some intermediate products or metabolic enzymes of glucose metabolism reprogramming can be used as molecular targets for early detection and evaluation of staging and prognosis of bladder cancer. Clear cell renal cell carcinoma (CCRCC) is a representative tumor of metabolic reprogramming in urinary system tumors. The metabolic reprogramming features of ccRCC include altered metabolic pathways associated with VHL gene inactivation and *Ras-PI3K-AKT-mTOR* pathway activation, involving aerobic glycolysis, fatty acid metabolism, and tryptophan, glutamine, and arginine utilization. These metabolic change biomarkers for early diagnosis offer new treatment ideas. Prostate cancer cells are more dependent on FAO pathways for metabolic reprogramming to obtain energy substances, which are closely related to androgens. Therefore, targeting lipid metabolic pathways and combined androgen deprivation therapy are major trends in the treatment of prostate cancer.

Metabolic reprogramming is central to the progression of urologic cancers and provides a window of opportunity for therapeutic intervention. Targeting metabolic pathways, either alone or in combination with other therapies such as immunotherapy, holds great potential for improving outcomes in these cancers. Clinical trials are currently investigating various metabolic inhibitors, and with a better understanding of metabolic profiles in individual tumors, more personalized treatments may be on the horizon.

### Future research directions and potential for clinical application

The metabolic reprogramming of urological tumors plays a crucial role in tumor growth, drug resistance, and immune escape. Future research will mainly focus on metabolic targeted therapy, metabolic immune interactions, tumor microenvironment regulation, and the combination of metabolomics and precision medicine. In metabolic targeted therapy, targeting key metabolic enzymes (such as FASN, SCD1, and ACC) and lipid and glucose metabolic pathways can inhibit tumor growth and be combined with immune checkpoint inhibitors and targeted therapy to form personalized treatment plans. In terms of metabolic immune interactions, studying how metabolism affects immune cell function, such as T cell depletion and macrophage polarization, and enhances the effectiveness of immunotherapy by regulating metabolism in the tumor microenvironment. In addition, research on the regulation of tumor microenvironment metabolism focuses on the acidic microenvironment and lactate metabolism to develop new drugs, such as anti-angiogenesis and lactate dehydrogenase inhibitors. At present, multiple omics technologies, such as metabolomics, proteomics, and genomics, can be used to comprehensively analyze the metabolic characteristics of urinary tract tumors and identify new therapeutic targets. If applied in clinical practice, personalized treatment plans can be customized by analyzing patients' metabolic characteristics, and early screening and personalized treatment strategies can be developed. These studies not only contribute to understanding the biological mechanisms of urological tumors but also provide new targets and strategies for future treatments.

## Figures and Tables

**Figure 1:**
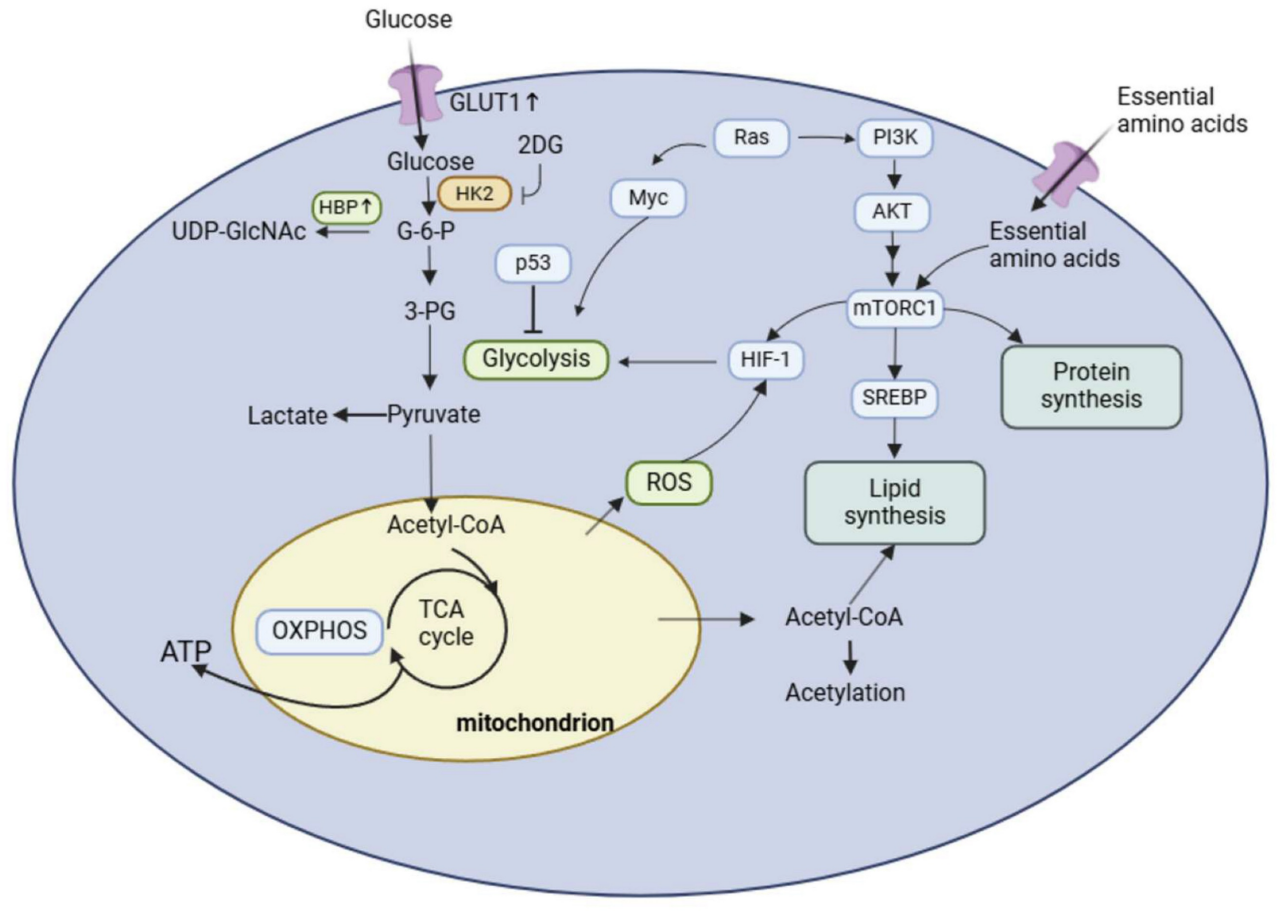
Metabolic Reprogramming in Bladder Cancer. Glycolysis converts glucose into lactic acid. Pyruvate enters the TCA cycle to produce energy in the mitochondria to support cell survival and to produce ROS. Amino acid metabolism, which provides relevant intermediates for cell function, regulates lipid metabolism via the mTOR signaling pathway.

**Figure 2:**
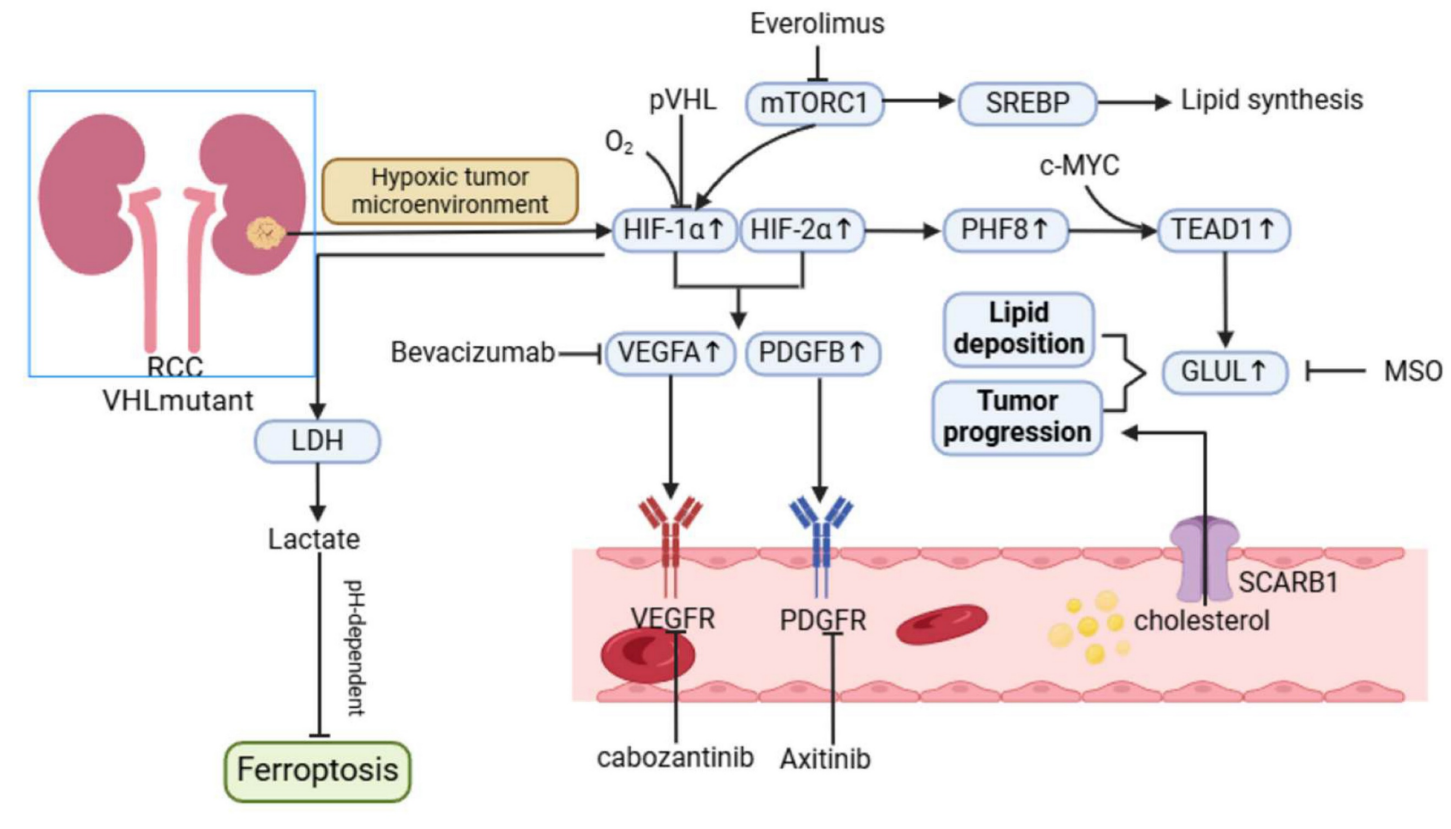
Metabolic reprogramming of renal cell carcinoma. The hypoxic tumor microenvironment and mTOR signaling upregulate the HIF pathway, promoting lipid deposition and tumor progression through the PHF8-GLUL axis. 2. Promote tumor angiogenesis and metastasis by promoting the expression of VEGFA, PDGFB, and other proteins. 3. Generate lactic acid through the Warburg effect and inhibit pH-dependent iron death through lactate dehydrogenase (LDH).

**Figure 3:**
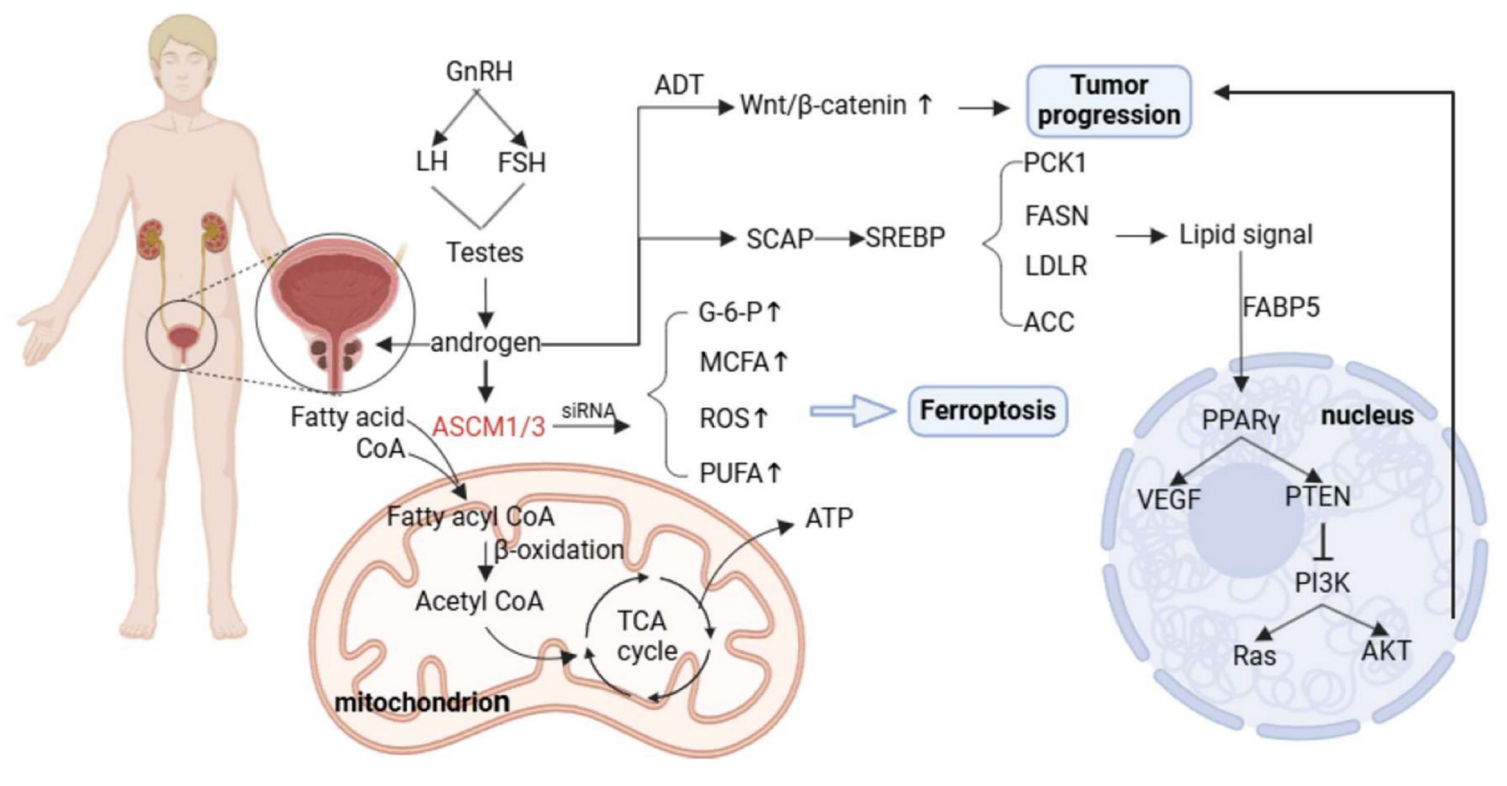
Metabolic reprogramming of prostate cancer. Androgens produced through the hypothalamic pituitary gonadal axis act on AR. AR can directly regulate ACSM1/3 and promote the entry of fatty acids into mitochondria for β-oxidation. Silencing ACSM1/3 can lead to ferroptosis of prostate cancer cells. AR signals can regulate SREBP expression and promote lipid deposition. After ADT therapy, the Wnt/β - catenin pathway is reflexively upregulated, leading to continued tumor progression.

**Table 1:**
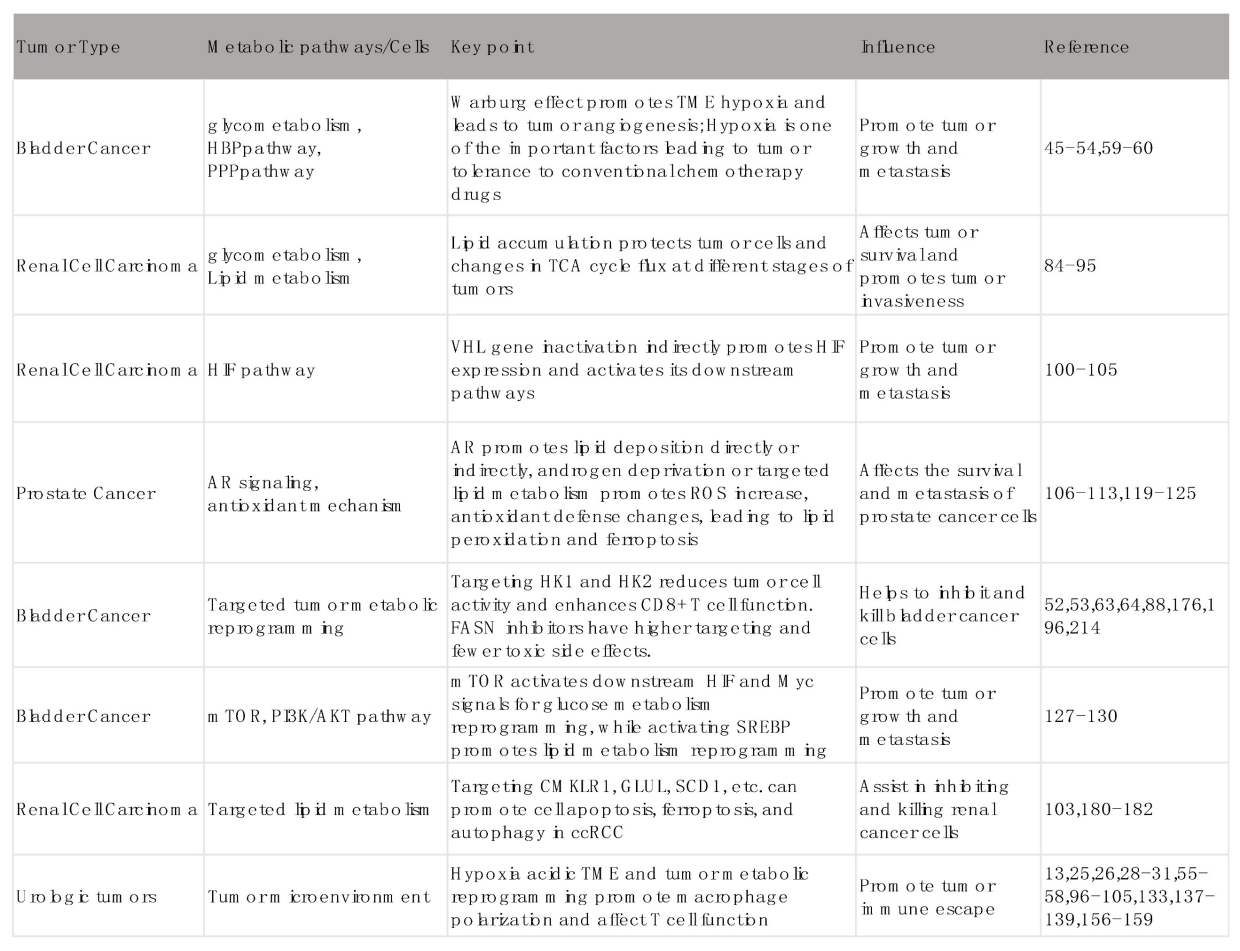
Key pathways of metabolic reprogramming in urinary tumors and their influence.

**Table 2:** Current progress in clinical development of targeted drugs for metabolic reprogramming.

Metabolic Pathway	Target	Representative drug / modality	Representative NCT	Clinical development stage
Lipid metabolism (fatty acid synthesis)	FASN	TVB-2640 (FASN inhibitor)	NCT05743621	Phase I (combination trials in mCRPC and other tumors)
Amino acid / glutamine metabolism	GLS1	Telaglenastat (CB-839)	NCT03875313	Phase I/II
Glycolysis / lactate transport	MCT1 (SLC16A1)	AZD3965 (MCT1 inhibitor)	NCT01791595	Phase I completed (dose-escalation in advanced cancers)
Glycolysis regulation	PFKFB3	PFK158 (PFKFB3 inhibitor)	NCT02044861	Phase I
Lipid signaling / lipid hydrolysis	MAGL	LEI-515 and other reversible MAGL inhibitors (preclinical)	Preclinical / early-stage; no widely registered late-phase oncology NCTs yet	Preclinical to exploratory; reversible MAGL inhibitors reported (LEI-515)
Arginine metabolism	Arginine deprivation (ASS1 deficiency)	ADI-PEG20 (pegylated arginine deiminase)	Multiple trials (e.g., NCT02709512 protocol)	Phase II/III in some indications (mesothelioma, sarcoma); explored in ASS1-deficient bladder cancers (early trials)
Tryptophan - kynurenine (immune metabolism)	IDO1	Epacadostat and other IDO1 inhibitors	Multiple trials (e.g., NCT02752074 was phase III melanoma)	Phase I-III historically; several negative phase III results led to reassessment; still under exploration in biomarker-selected combos
Fatty acid desaturation / synthesis	SCD1 / ACC	SCD1/ACC inhibitors (multiple preclinical and early clinical candidates)	Mostly preclinical/early-stage; some ACC inhibitors have entered Phase I for metabolic diseases and oncology	Preclinical to early clinical
Immune metabolism (Indirectly regulate T cell glucose metabolism)	KLK2 × CD3 bispecific	Pasritamig(JNJ-78278343)	NCT04898634	Phase I
Protein biosynthesis	MetAP2	APL-1202	NCT04813107	Phase I/II

## Abbreviations

TCA: Tricarboxylic acid cycle
ICI: Immune checkpoint inhibitor
RCC: Renal cell carcinoma
GLUT: Glucose transporter
IGF: Insulin-like growth factor
AR: Androgen receptor
PKM2: Pyruvate kinase M2
LDHA: Lactate dehydrogenase A
HIF: Hypoxia-inducible factor
ROS: Reactive oxygen species
ECM: Extracellular matrix
HK2: Hexokinase 2
2-DG: 2-deoxy-D-glucose
PPP: Pentose phosphate pathway
ACSS2: Acetyl-CoA synthetase 2
SREBPs: Sterol-regulatory element binding proteins
ATGL: Adipose triglyceride lipase
mTOR: mammalian target of rapamycin
ox-LDL: Oxidized low-density lipoprotein
TAM: tumor-associated macrophage
PPAR: Peroxisome proliferator-activated receptors
CSCs: Cancer Stem Cells
FAO: Fatty acid oxidation
FASN: Fatty acid synthase
ACC: acetyl-CoA carboxylase
PDK1: Pyruvate dehydrogenase kinase 1
CAFs: Cancer-associated fibroblasts
ADT: Androgen deprivation therapy
HAFs: Hypoxia-associated factors
VEGF: Vascular endothelial growth factor
SCAP: SREBP cleavage—activating protein
PFK: Phosphofructokinase
ADPGK: ADP-dependent glucokinase
OXPHOS: Oxidative phosphorylation
TANs: Tumor-associated neutrophils
FABP: Fatty acid-binding protein
MGLL: Monoglyceride lipase

## References

[1] Flaig TW, Spiess PE, Agarwal N, Bangs R, Boorjian SA, Buyyounouski MK (2020). Bladder Cancer, Version 3.2020, NCCN Clinical Practice Guidelines in Oncology. Journal of the National Comprehensive Cancer Network: JNCCN.

[2] Li W, Shangguan W, Huang W, Zhao J, Zhu Y, Xie M (2025). Gut Parabacteroides distasonis-derived Indole-3-Acetic Acid Promotes Phospholipid Remodeling and Enhances Ferroptosis Sensitivity via the AhR-FASN Axis in Bladder Cancer. Adv Sci (Weinh).

[3] Cheng B, Wu J, Chen K, Li W, Yang J, Shangguan W (2025). Association of 5α-reductase inhibitor prescription with immunotherapy efficacy in metastatic renal cell carcinoma: a multicenter retrospective analysis. J Immunother Cancer.

[4] Kamat AM, Hahn NM, Efstathiou JA, Lerner SP, Malmström PU, Choi W (2016). Bladder cancer. Lancet (London, England).

[5] Corgna E, Betti M, Gatta G, Roila F, De Mulder PH (2007). Renal cancer. Critical reviews in oncology/hematology.

[6] Capitanio U, Terrone C, Antonelli A, Minervini A, Volpe A, Furlan M (2015). Nephron-sparing techniques independently decrease the risk of cardiovascular events relative to radical nephrectomy in patients with a T1a-T1b renal mass and normal preoperative renal function. European urology.

[7] Huang WC, Elkin EB, Levey AS, Jang TL, Russo P (2009). Partial nephrectomy versus radical nephrectomy in patients with small renal tumors-is there a difference in mortality and cardiovascular outcomes?. The Journal of urology.

[8] Rizzo A, Santoni M, Mollica V, Fiorentino M, Brandi G, Massari F (2022). Microbiota and prostate cancer. Seminars in cancer biology.

[9] Wang G, Zhao D, Spring DJ, DePinho RA (2018). Genetics and biology of prostate cancer. Genes & development.

[10] Prostate cancer Nature reviews Disease primers. 2021; 7: 8.

[11] Cheng B, Yang J, Huang W, Luo T, Li L, Wu J (2025). Regulation of macrophage plasticity by circCCDC719-13 through HSP90 inhibition suppresses prostate cancer progression and metastasis: a translational study. Int J Surg.

[12] Cheng B, Luo T, Wu Y, Hu J, Yang C, Wu J (2025). Urinary exosomal FAM153C-RPL19 chimeric RNA as a diagnostic and prognostic biomarker for prostate cancer in Chinese patients. Cancer Lett.

[13] Paller CJ, Antonarakis ES (2013). Management of biochemically recurrent prostate cancer after local therapy: evolving standards of care and new directions. Clinical advances in hematology & oncology: H&O.

[14] Aytes A, Mitrofanova A, Lefebvre C, Alvarez MJ, Castillo-Martin M, Zheng T (2014). Cross-species regulatory network analysis identifies a synergistic interaction between FOXM1 and CENPF that drives prostate cancer malignancy. Cancer cell.

[15] Mitrofanova A, Aytes A, Zou M, Shen MM, Abate-Shen C, Califano A (2015). Predicting Drug Response in Human Prostate Cancer from Preclinical Analysis of In Vivo Mouse Models. Cell reports.

[16] Beer TM, Kwon ED, Drake CG, Fizazi K, Logothetis C, Gravis G (2017). Randomized, Double-Blind, Phase III Trial of Ipilimumab Versus Placebo in Asymptomatic or Minimally Symptomatic Patients With Metastatic Chemotherapy-Naive Castration-Resistant Prostate Cancer. Journal of clinical oncology: official journal of the American Society of Clinical Oncology.

[17] Xia L, Oyang L, Lin J, Tan S, Han Y, Wu N (2021). The cancer metabolic reprogramming and immune response. Molecular cancer.

[18] Massari F, Ciccarese C, Santoni M, Iacovelli R, Mazzucchelli R, Piva F (2016). Metabolic phenotype of bladder cancer. Cancer treatment reviews.

[19] Li M, Yu J, Ju L, Wang Y, Jin W, Zhang R (2024). USP43 stabilizes c-Myc to promote glycolysis and metastasis in bladder cancer. Cell death & disease.

[20] Wang JZ, Zhu W, Han J, Yang X, Zhou R, Lu HC (2021). The role of the HIF-1α/ALYREF/PKM2 axis in glycolysis and tumorigenesis of bladder cancer. Cancer communications (London, England).

[21] Bobulescu IA, Pop LM, Mani C, Turner K, Rivera C, Khatoon S (2021). Renal Lipid Metabolism Abnormalities in Obesity and Clear Cell Renal Cell Carcinoma. Metabolites.

[22] Tan SK, Hougen HY, Merchan JR, Gonzalgo ML, Welford SM (2023). Fatty acid metabolism reprogramming in ccRCC: mechanisms and potential targets. Nature reviews Urology.

[23] Desai K, McManus JM, Sharifi N (2021). Hormonal Therapy for Prostate Cancer. Endocrine reviews.

[24] Dai C, Dehm SM, Sharifi N (2023). Targeting the Androgen Signaling Axis in Prostate Cancer. Journal of clinical oncology: official journal of the American Society of Clinical Oncology.

[25] Li B, Cheng B, Huang H, Huang S, Yu S, Li Z (2024). Darolutamide-mediated phospholipid remodeling induces ferroptosis through the SREBP1-FASN axis in prostate cancer. Int J Biol Sci.

[26] Fendt SM, Frezza C, Erez A (2020). Targeting Metabolic Plasticity and Flexibility Dynamics for Cancer Therapy. Cancer discovery.

[27] Warburg O, Wind F, Negelein E (1927). THE METABOLISM OF TUMORS IN THE BODY. The Journal of general physiology.

[28] Altman BJ, Stine ZE, Dang CV (2016). From Krebs to clinic: glutamine metabolism to cancer therapy. Nature reviews Cancer.

[29] Masoud GN, Li W (2015). HIF-1α pathway: role, regulation and intervention for cancer therapy. Acta pharmaceutica Sinica B.

[30] Wang J, Xu W, Wang B, Lin G, Wei Y, Abudurexiti M (2020). GLUT1 is an AR target contributing to tumor growth and glycolysis in castration-resistant and enzalutamide-resistant prostate cancers. Cancer letters.

[31] Watson MJ, Vignali PDA, Mullett SJ, Overacre-Delgoffe AE, Peralta RM, Grebinoski S (2021). Metabolic support of tumour-infiltrating regulatory T cells by lactic acid. Nature.

[32] Cheng B, Li L, Wu Y, Luo T, Tang C, Wang Q (2023). The key cellular senescence related molecule RRM2 regulates prostate cancer progression and resistance to docetaxel treatment. Cell Biosci.

[33] Li Z, Zhang H (2016). Reprogramming of glucose, fatty acid and amino acid metabolism for cancer progression. Cellular and molecular life sciences: CMLS.

[34] Gao P, Tchernyshyov I, Chang TC, Lee YS, Kita K, Ochi T (2009). c-Myc suppression of miR-23a/b enhances mitochondrial glutaminase expression and glutamine metabolism. Nature.

[35] Wise DR, DeBerardinis RJ, Mancuso A, Sayed N, Zhang XY, Pfeiffer HK (2008). Myc regulates a transcriptional program that stimulates mitochondrial glutaminolysis and leads to glutamine addiction. Proceedings of the National Academy of Sciences of the United States of America.

[36] Le A, Lane AN, Hamaker M, Bose S, Gouw A, Barbi J (2012). Glucose-independent glutamine metabolism via TCA cycling for proliferation and survival in B cells. Cell metabolism.

[37] Hensley CT, Wasti AT, DeBerardinis RJ (2013). Glutamine and cancer: cell biology, physiology, and clinical opportunities. The Journal of clinical investigation.

[38] van Dam L, Korolev N, Nordenskiöld L (2002). Polyamine-nucleic acid interactions and the effects on structure in oriented DNA fibers. Nucleic acids research.

[39] Lieu EL, Nguyen T, Rhyne S, Kim J (2020). Amino acids in cancer. Experimental & molecular medicine.

[40] Röhrig F, Schulze A (2016). The multifaceted roles of fatty acid synthesis in cancer. Nature reviews Cancer.

[41] Bian X, Liu R, Meng Y, Xing D, Xu D, Lu Z (2021). Lipid metabolism and cancer. The Journal of experimental medicine.

[42] Rysman E, Brusselmans K, Scheys K, Timmermans L, Derua R, Munck S (2010). De novo lipogenesis protects cancer cells from free radicals and chemotherapeutics by promoting membrane lipid saturation. Cancer research.

[43] Yadav D, Yadav A, Bhattacharya S, Dagar A, Kumar V, Rani R (2024). GLUT and HK: Two primary and essential key players in tumor glycolysis. Seminars in cancer biology.

[44] Lawrence MS, Stojanov P, Mermel CH, Robinson JT, Garraway LA, Golub TR (2014). Discovery and saturation analysis of cancer genes across 21 tumour types. Nature.

[45] Zhang L, Zhan Y, Li L, Deng H, Wang J, Zhu Z (2022). CircOMA1 promotes tumour growth and metastasis of bladder cancer by modulating IGF-IR/MAPK/EMT pathway. Clinical and translational medicine.

[46] Shi R, Tang YQ, Miao H (2020). Metabolism in tumor microenvironment: Implications for cancer immunotherapy. MedComm.

[47] Hao X, Ren Y, Feng M, Wang Q, Wang Y (2021). Metabolic reprogramming due to hypoxia in pancreatic cancer: Implications for tumor formation, immunity, and more. Biomedicine & pharmacotherapy = Biomedecine & pharmacotherapie.

[48] Ma J, Huang L, Hu D, Zeng S, Han Y, Shen H (2021). The role of the tumor microbe microenvironment in the tumor immune microenvironment: bystander, activator, or inhibitor?. Journal of experimental & clinical cancer research: CR.

[49] O'Neill LA, Kishton RJ, Rathmell J (2016). A guide to immunometabolism for immunologists. Nature reviews Immunology.

[50] Geiger R, Rieckmann JC, Wolf T, Basso C, Feng Y, Fuhrer T (2016). L-Arginine Modulates T Cell Metabolism and Enhances Survival and Anti-tumor Activity. Cell.

[51] Li X, Peng X, Zhang C, Bai X, Li Y, Chen G (2022). Bladder Cancer-Derived Small Extracellular Vesicles Promote Tumor Angiogenesis by Inducing HBP-Related Metabolic Reprogramming and SerRS O-GlcNAcylation in Endothelial Cells. Advanced science (Weinheim, Baden-Wurttemberg, Germany).

[52] Wu S, Ou T, Xing N, Lu J, Wan S, Wang C (2019). Whole-genome sequencing identifies ADGRG6 enhancer mutations and FRS2 duplications as angiogenesis-related drivers in bladder cancer. Nature communications.

[53] Reinfeld BI, Madden MZ, Wolf MM, Chytil A, Bader JE, Patterson AR (2021). Cell-programmed nutrient partitioning in the tumour microenvironment. Nature.

[54] Hosios AM, Manning BD (2021). Cancer Signaling Drives Cancer Metabolism: AKT and the Warburg Effect. Cancer research.

[55] De Jesus A, Keyhani-Nejad F, Pusec CM, Goodman L, Geier JA, Stoolman JS (2022). Hexokinase 1 cellular localization regulates the metabolic fate of glucose. Mol Cell.

[56] Coussement P, Bauwens D, Peters G, Maertens J, De Mey M (2020). Mapping and refactoring pathway control through metabolic and protein engineering: The hexosamine biosynthesis pathway. Biotechnology advances.

[57] Sharma NS, Gupta VK, Garrido VT, Hadad R, Durden BC, Kesh K (2020). Targeting tumor-intrinsic hexosamine biosynthesis sensitizes pancreatic cancer to anti-PD1 therapy. The Journal of clinical investigation.

[58] Afonso J, Gonçalves C, Costa M, Ferreira D, Santos L, Longatto-Filho A (2023). Glucose Metabolism Reprogramming in Bladder Cancer: Hexokinase 2 (HK2) as Prognostic Biomarker and Target for Bladder Cancer Therapy. Cancers.

[59] Afonso J, Santos LL, Longatto-Filho A, Baltazar F (2020). Competitive glucose metabolism as a target to boost bladder cancer immunotherapy. Nature reviews Urology.

[60] Ferreira E, Ferreira D, Relvas-Santos M, Freitas R, Soares J, Azevedo R (2024). Aberrantly Glycosylated GLUT1 as a Poor Prognosis Marker in Aggressive Bladder Cancer. International journal of molecular sciences.

[61] Rouprêt M, Seisen T, Birtle AJ, Capoun O, Compérat EM, Dominguez-Escrig JL (2023). European Association of Urology Guidelines on Upper Urinary Tract Urothelial Carcinoma: 2023 Update. European urology.

[62] Yang C, Deng X, Tang Y, Tang H, Xia C (2024). Natural products reverse cisplatin resistance in the hypoxic tumor microenvironment. Cancer letters.

[63] Duan M, Leng S, Mao P (2024). Cisplatin in the era of PARP inhibitors and immunotherapy. Pharmacology & therapeutics.

[64] Zhang C, Xu C, Gao X, Yao Q (2022). Platinum-based drugs for cancer therapy and anti-tumor strategies. Theranostics.

[65] Li F, Zhang H, Huang Y, Li D, Zheng Z, Xie K (2024). Single-cell transcriptome analysis reveals the association between histone lactylation and cisplatin resistance in bladder cancer. Drug resistance updates: reviews and commentaries in antimicrobial and anticancer chemotherapy.

[66] Cheng B, Li L, Luo T, Wang Q, Luo Y, Bai S (2024). Single-cell deconvolution algorithms analysis unveils autocrine IL11-mediated resistance to docetaxel in prostate cancer via activation of the JAK1/STAT4 pathway. J Exp Clin Cancer Res.

[67] Shigeta K, Hasegawa M, Hishiki T, Naito Y, Baba Y, Mikami S (2023). IDH2 stabilizes HIF-1α-induced metabolic reprogramming and promotes chemoresistance in urothelial cancer. The EMBO journal.

[68] Wen H, Lee S, Zhu WG, Lee OJ, Yun SJ, Kim J (2019). Glucose-derived acetate and ACSS2 as key players in cisplatin resistance in bladder cancer. Biochimica et biophysica acta Molecular and cell biology of lipids.

[69] Teleka S, Häggström C, Nagel G, Bjørge T, Manjer J, Ulmer H (2018). Risk of bladder cancer by disease severity in relation to metabolic factors and smoking: A prospective pooled cohort study of 800,000 men and women. International journal of cancer.

[70] Piyarathna DWB, Rajendiran TM, Putluri V, Vantaku V, Soni T, von Rundstedt FC (2018). Distinct Lipidomic Landscapes Associated with Clinical Stages of Urothelial Cancer of the Bladder. European urology focus.

[71] Chandrasekaran P, Weiskirchen R (2024). The Role of SCAP/SREBP as Central Regulators of Lipid Metabolism in Hepatic Steatosis. International journal of molecular sciences.

[72] Zhang Y, Xiao B, Liu Y, Wu S, Xiang Q, Xiao Y (2024). Roles of PPAR activation in cancer therapeutic resistance: Implications for combination therapy and drug development. European journal of pharmacology.

[73] Jia LH, Hu MD, Liu Y, Xiong X, Wang WJ, Wang JG (2019). HSDL2 Promotes Bladder Cancer Growth In Vitro and In Vivo. International journal of medical sciences.

[74] Jiao F, Sun H, Yang Q, Sun H, Wang Z, Liu M (2020). Identification of FADS1 Through Common Gene Expression Profiles for Predicting Survival in Patients with Bladder Cancer. Cancer management and research.

[75] Horton JD, Shah NA, Warrington JA, Anderson NN, Park SW, Brown MS (2003). Combined analysis of oligonucleotide microarray data from transgenic and knockout mice identifies direct SREBP target genes. Proceedings of the National Academy of Sciences of the United States of America.

[76] Ricoult SJ, Yecies JL, Ben-Sahra I, Manning BD (2016). Oncogenic PI3K and K-Ras stimulate de novo lipid synthesis through mTORC1 and SREBP. Oncogene.

[77] Kamphorst JJ, Cross JR, Fan J, de Stanchina E, Mathew R, White EP (2013). Hypoxic and Ras-transformed cells support growth by scavenging unsaturated fatty acids from lysophospholipids. Proceedings of the National Academy of Sciences of the United States of America.

[78] Yang L, Sun J, Li M, Long Y, Zhang D, Guo H (2021). Oxidized Low-Density Lipoprotein Links Hypercholesterolemia and Bladder Cancer Aggressiveness by Promoting Cancer Stemness. Cancer research.

[79] Moore KJ, Sheedy FJ, Fisher EA (2013). Macrophages in atherosclerosis: a dynamic balance. Nature reviews Immunology.

[80] Nègre-Salvayre A, Augé N, Camaré C, Bacchetti T, Ferretti G, Salvayre R (2017). Dual signaling evoked by oxidized LDLs in vascular cells. Free radical biology & medicine.

[81] Zeng Y, Luo Y, Zhao K, Liu S, Wu K, Wu Y (2024). m6A-Mediated Induction of 7-Dehydrocholesterol Reductase Stimulates Cholesterol Synthesis and cAMP Signaling to Promote Bladder Cancer Metastasis. Cancer research.

[82] Xiong Q, Feng D, Wang Z, Ying Y, Xu C, Wei Q (2022). Fatty Acid Synthase Is the Key Regulator of Fatty Acid Metabolism and Is Related to Immunotherapy in Bladder Cancer. Frontiers in immunology.

[83] Song T, He K, Ning J, Li W, Xu T, Yu W (2022). Evaluation of aliphatic acid metabolism in bladder cancer with the goal of guiding therapeutic treatment. Frontiers in oncology.

[84] 涂生 王, 王行环 (2024). 膀胱癌脂质代谢的研究现状及展望. 中华实验外科杂志. 2024.

[85] Ng K, Stenzl A, Sharma A, Vasdev N (2021). Urinary biomarkers in bladder cancer: A review of the current landscape and future directions. Urologic oncology.

[86] Maas M, Todenhöfer T, Black PC (2023). Urine biomarkers in bladder cancer - current status and future perspectives. Nature reviews Urology.

[87] Costa VL, Henrique R, Danielsen SA, Duarte-Pereira S, Eknaes M, Skotheim RI (2010). Three epigenetic biomarkers, GDF15, TMEFF2, and VIM, accurately predict bladder cancer from DNA-based analyses of urine samples. Clinical cancer research: an official journal of the American Association for Cancer Research.

[88] van Kessel KE, Beukers W, Lurkin I, Ziel-van der Made A, van der Keur KA, Boormans JL (2017). Validation of a DNA Methylation-Mutation Urine Assay to Select Patients with Hematuria for Cystoscopy. The Journal of urology.

[89] van Kessel KEM, de Jong JJ, Ziel-van der Made ACJ, Roshani H, Haensel SM, Wolterbeek JH (2020). A Urine Based Genomic Assay to Triage Patients with Hematuria for Cystoscopy. The Journal of urology.

[90] O'Sullivan P, Sharples K, Dalphin M, Davidson P, Gilling P, Cambridge L (2012). A multigene urine test for the detection and stratification of bladder cancer in patients presenting with hematuria. The Journal of urology.

[91] Wallace E, Higuchi R, Satya M, McCann L, Sin MLY, Bridge JA (2018). Development of a 90-Minute Integrated Noninvasive Urinary Assay for Bladder Cancer Detection. The Journal of urology.

[92] Du Y, Wang Q, Zhang X, Wang X, Qin C, Sheng Z (2017). Lysophosphatidylcholine acyltransferase 1 upregulation and concomitant phospholipid alterations in clear cell renal cell carcinoma. Journal of experimental & clinical cancer research: CR.

[93] Zhao Z, Liu Y, Liu Q, Wu F, Liu X, Qu H (2019). The mRNA Expression Signature and Prognostic Analysis of Multiple Fatty Acid Metabolic Enzymes in Clear Cell Renal Cell Carcinoma. Journal of Cancer.

[94] Teng L, Chen Y, Cao Y, Wang W, Xu Y, Wang Y (2018). Overexpression of ATP citrate lyase in renal cell carcinoma tissues and its effect on the human renal carcinoma cells in vitro. Oncology letters.

[95] Yuan Y, Yang X, Li Y, Liu Q, Wu F, Qu H (2020). Expression and prognostic significance of fatty acid synthase in clear cell renal cell carcinoma. Pathology, research and practice.

[96] Falchook G, Infante J, Arkenau HT, Patel MR, Dean E, Borazanci E (2021). First-in-human study of the safety, pharmacokinetics, and pharmacodynamics of first-in-class fatty acid synthase inhibitor TVB-2640 alone and with a taxane in advanced tumors. EClinicalMedicine.

[97] Mwaikambo BR, Yang C, Chemtob S, Hardy P (2009). Hypoxia up-regulates CD36 expression and function via hypoxia-inducible factor-1- and phosphatidylinositol 3-kinase-dependent mechanisms. The Journal of biological chemistry.

[98] Riscal R, Bull CJ, Mesaros C, Finan JM, Carens M, Ho ES (2021). Cholesterol Auxotrophy as a Targetable Vulnerability in Clear Cell Renal Cell Carcinoma. Cancer discovery.

[99] Garcia-Bermudez J, Baudrier L, Bayraktar EC, Shen Y, La K, Guarecuco R (2019). Squalene accumulation in cholesterol auxotrophic lymphomas prevents oxidative cell death. Nature.

[100] Kim JW, Tchernyshyov I, Semenza GL, Dang CV (2006). HIF-1-mediated expression of pyruvate dehydrogenase kinase: a metabolic switch required for cellular adaptation to hypoxia. Cell metabolism.

[101] Wettersten HI, Hakimi AA, Morin D, Bianchi C, Johnstone ME, Donohoe DR (2015). Grade-Dependent Metabolic Reprogramming in Kidney Cancer Revealed by Combined Proteomics and Metabolomics Analysis. Cancer research.

[102] Bezwada D, Perelli L, Lesner NP, Cai L, Brooks B, Wu Z (2024). Mitochondrial complex I promotes kidney cancer metastasis. Nature.

[103] Chang K, Chen Y, Zhang X, Zhang W, Xu N, Zeng B (2023). DPP9 Stabilizes NRF2 to Suppress Ferroptosis and Induce Sorafenib Resistance in Clear Cell Renal Cell Carcinoma. Cancer research.

[104] Rabinowitz JD, Enerbäck S (2020). Lactate: the ugly duckling of energy metabolism. Nature metabolism.

[105] Colegio OR, Chu NQ, Szabo AL, Chu T, Rhebergen AM, Jairam V (2014). Functional polarization of tumour-associated macrophages by tumour-derived lactic acid. Nature.

[106] Palmieri EM, Menga A, Martín-Pérez R, Quinto A, Riera-Domingo C, De Tullio G (2017). Pharmacologic or Genetic Targeting of Glutamine Synthetase Skews Macrophages toward an M1-like Phenotype and Inhibits Tumor Metastasis. Cell reports.

[107] Fu W, Zhao H, Liu Y, Nie H, Gao B, Yin F (2022). Exosomes Derived from Cancer-Associated Fibroblasts Regulate Cell Progression in Clear-Cell Renal-Cell Carcinoma. Nephron.

[108] Gossage L, Eisen T (2010). Alterations in VHL as potential biomarkers in renal-cell carcinoma. Nature reviews Clinical oncology.

[109] Choueiri TK, Kaelin WG Jr (2020). Targeting the HIF2-VEGF axis in renal cell carcinoma. Nature medicine.

[110] Yang Z, Su W, Wei X, Qu S, Zhao D, Zhou J (2023). HIF-1α drives resistance to ferroptosis in solid tumors by promoting lactate production and activating SLC1A1. Cell reports.

[111] Peng S, Wang Z, Tang P, Wang S, Huang Y, Xie Q (2023). PHF8-GLUL axis in lipid deposition and tumor growth of clear cell renal cell carcinoma. Science advances.

[112] Li Z, Bao S, Wu Q, Wang H, Eyler C, Sathornsumetee S (2009). Hypoxia-inducible factors regulate tumorigenic capacity of glioma stem cells. Cancer cell.

[113] Heddleston JM, Li Z, Lathia JD, Bao S, Hjelmeland AB, Rich JN (2010). Hypoxia inducible factors in cancer stem cells. British journal of cancer.

[114] Butler LM, Centenera MM, Swinnen JV (2016). Androgen control of lipid metabolism in prostate cancer: novel insights and future applications. Endocrine-related cancer.

[115] Cheng B, Huang H (2023). Expanding horizons in overcoming therapeutic resistance in castration-resistant prostate cancer: targeting the androgen receptor-regulated tumor immune microenvironment. Cancer Biol Med.

[116] Shrestha RK, Nassar ZD, Hanson AR, Iggo R, Townley SL, Dehairs J (2024). ACSM1 and ACSM3 Regulate Fatty Acid Metabolism to Support Prostate Cancer Growth and Constrain Ferroptosis. Cancer research.

[117] Swinnen JV, Ulrix W, Heyns W, Verhoeven G (1997). Coordinate regulation of lipogenic gene expression by androgens: evidence for a cascade mechanism involving sterol regulatory element binding proteins. Proceedings of the National Academy of Sciences of the United States of America.

[118] Bader DA, McGuire SE (2020). Tumour metabolism and its unique properties in prostate adenocarcinoma. Nature reviews Urology.

[119] Carbonetti G, Wilpshaar T, Kroonen J, Studholme K, Converso C, d'Oelsnitz S (2019). FABP5 coordinates lipid signaling that promotes prostate cancer metastasis. Scientific reports.

[120] Ahmad I, Mui E, Galbraith L, Patel R, Tan EH, Salji M (2016). Sleeping Beauty screen reveals Pparg activation in metastatic prostate cancer. Proceedings of the National Academy of Sciences of the United States of America.

[121] Robinson D, Van Allen EM, Wu YM, Schultz N, Lonigro RJ, Mosquera JM (2015). Integrative clinical genomics of advanced prostate cancer. Cell.

[122] Lee E, Ha S, Logan SK (2015). Divergent Androgen Receptor and Beta-Catenin Signaling in Prostate Cancer Cells. PloS one.

[123] Gonzalez-Menendez P, Hevia D, Alonso-Arias R, Alvarez-Artime A, Rodriguez-Garcia A, Kinet S (2018). GLUT1 protects prostate cancer cells from glucose deprivation-induced oxidative stress. Redox biology.

[124] Xu H, Li YF, Yi XY, Zheng XN, Yang Y, Wang Y (2023). ADP-dependent glucokinase controls metabolic fitness in prostate cancer progression. Military Medical Research.

[125] Kumagai S, Koyama S, Itahashi K, Tanegashima T, Lin YT, Togashi Y (2022). Lactic acid promotes PD-1 expression in regulatory T cells in highly glycolytic tumor microenvironments. Cancer cell.

[126] Ippolito L, Morandi A, Giannoni E, Chiarugi P (2019). Lactate: A Metabolic Driver in the Tumour Landscape. Trends in biochemical sciences.

[127] Wang Y, Gapstur SM, Newton CC, McCullough ML, Pollak MN, Campbell PT (2022). Biomarkers of Glucose Homeostasis and Inflammation with Risk of Prostate Cancer: A Case-Cohort Study. Cancer epidemiology, biomarkers & prevention: a publication of the American Association for Cancer Research, cosponsored by the American Society of Preventive Oncology.

[128] Tam NN, Gao Y, Leung YK, Ho SM (2003). Androgenic regulation of oxidative stress in the rat prostate: involvement of NAD(P)H oxidases and antioxidant defense machinery during prostatic involution and regrowth. The American journal of pathology.

[129] Bolduc JA, Collins JA, Loeser RF (2019). Reactive oxygen species, aging and articular cartilage homeostasis. Free radical biology & medicine.

[130] Shiota M, Sekino Y, Tsukahara S, Abe T, Kinoshita F, Imada K (2021). Gene amplification of YB-1 in castration-resistant prostate cancer in association with aberrant androgen receptor expression. Cancer science.

[131] Fan J, Fan Y, Wang X, Niu L, Duan L, Yang J (2019). PLCε regulates prostate cancer mitochondrial oxidative metabolism and migration via upregulation of Twist1. Journal of experimental & clinical cancer research: CR.

[132] Hasle N, Matreyek KA, Fowler DM (2019). The Impact of Genetic Variants on PTEN Molecular Functions and Cellular Phenotypes. Cold Spring Harbor perspectives in medicine.

[133] Battisti V, Maders LD, Bagatini MD, Reetz LG, Chiesa J, Battisti IE (2011). Oxidative stress and antioxidant status in prostate cancer patients: relation to Gleason score, treatment and bone metastasis. Biomedicine & pharmacotherapy = Biomedecine & pharmacotherapie.

[134] Lai W, Zhu W, Wu J, Huang J, Li X, Luo Y (2024). HJURP inhibits sensitivity to ferroptosis inducers in prostate cancer cells by enhancing the peroxidase activity of PRDX1. Redox biology.

[135] Glaviano A, Foo ASC, Lam HY, Yap KCH, Jacot W, Jones RH (2023). PI3K/AKT/mTOR signaling transduction pathway and targeted therapies in cancer. Molecular cancer.

[136] Menon S, Dibble CC, Talbott G, Hoxhaj G, Valvezan AJ, Takahashi H (2014). Spatial control of the TSC complex integrates insulin and nutrient regulation of mTORC1 at the lysosome. Cell.

[137] Lee J, Kim MS (2007). The role of GSK3 in glucose homeostasis and the development of insulin resistance. Diabetes research and clinical practice.

[138] Porstmann T, Santos CR, Griffiths B, Cully M, Wu M, Leevers S (2008). SREBP activity is regulated by mTORC1 and contributes to Akt-dependent cell growth. Cell metabolism.

[139] Yao Y, Suraokar M, Darnay BG, Hollier BG, Shaiken TE, Asano T (2013). BSTA promotes mTORC2-mediated phosphorylation of Akt1 to suppress expression of FoxC2 and stimulate adipocyte differentiation. Science signaling.

[140] Mossmann D, Park S, Hall MN (2018). mTOR signalling and cellular metabolism are mutual determinants in cancer. Nature reviews Cancer.

[141] Liu GY, Sabatini DM (2020). mTOR at the nexus of nutrition, growth, ageing and disease. Nature reviews Molecular cell biology.

[142] Simcox J, Lamming DW (2022). The central moTOR of metabolism. Developmental cell.

[143] Wang D, Xu C, Yang W, Chen J, Ou Y, Guan Y (2022). E3 ligase RNF167 and deubiquitinase STAMBPL1 modulate mTOR and cancer progression. Mol Cell.

[144] Li H, Wen X, Ren Y, Fan Z, Zhang J, He G (2024). Targeting PI3K family with small-molecule inhibitors in cancer therapy: current clinical status and future directions. Molecular cancer.

[145] Benita Y, Kikuchi H, Smith AD, Zhang MQ, Chung DC, Xavier RJ (2009). An integrative genomics approach identifies Hypoxia Inducible Factor-1 (HIF-1)-target genes that form the core response to hypoxia. Nucleic acids research.

[146] McNeill LA, Hewitson KS, Gleadle JM, Horsfall LE, Oldham NJ, Maxwell PH (2002). The use of dioxygen by HIF prolyl hydroxylase (PHD1). Bioorganic & medicinal chemistry letters.

[147] Bailey CM, Liu Y, Liu M, Du X, Devenport M, Zheng P (2022). Targeting HIF-1α abrogates PD-L1-mediated immune evasion in tumor microenvironment but promotes tolerance in normal tissues. The Journal of clinical investigation.

[148] Zhang Z, Wang D, Xu R, Li X, Wang Z, Zhang Y The Physiological Functions and Therapeutic Potential of Hypoxia-Inducible Factor-1α in Vascular Calcification. 2024; 14: 1592.

[149] Dang CV, O'Donnell KA, Zeller KI, Nguyen T, Osthus RC, Li F (2006). The c-Myc target gene network. Seminars in cancer biology.

[150] Dong Y, Tu R, Liu H, Qing G (2020). Regulation of cancer cell metabolism: oncogenic MYC in the driver's seat. Signal transduction and targeted therapy.

[151] Gouw AM, Margulis K, Liu NS, Raman SJ, Mancuso A, Toal GG (2019). The MYC Oncogene Cooperates with Sterol-Regulated Element-Binding Protein to Regulate Lipogenesis Essential for Neoplastic Growth. Cell metabolism.

[152] Cao Z, Fan-Minogue H, Bellovin DI, Yevtodiyenko A, Arzeno J, Yang Q (2011). MYC phosphorylation, activation, and tumorigenic potential in hepatocellular carcinoma are regulated by HMG-CoA reductase. Cancer research.

[153] Zhang H, Gao P, Fukuda R, Kumar G, Krishnamachary B, Zeller KI (2007). HIF-1 inhibits mitochondrial biogenesis and cellular respiration in VHL-deficient renal cell carcinoma by repression of C-MYC activity. Cancer cell.

[154] Du W, Zhang L, Brett-Morris A, Aguila B, Kerner J, Hoppel CL (2017). HIF drives lipid deposition and cancer in ccRCC via repression of fatty acid metabolism. Nature communications.

[155] Mascaraque M, Courtois S, Royo-García A, Barneda D, Stoian AM, Villaoslada I (2024). Fatty acid oxidation is critical for the tumorigenic potential and chemoresistance of pancreatic cancer stem cells. Journal of translational medicine.

[156] Olzmann JA, Carvalho P (2019). Dynamics and functions of lipid droplets. Nature reviews Molecular cell biology.

[157] García-Prat L, Martínez-Vicente M, Perdiguero E, Ortet L, Rodríguez-Ubreva J, Rebollo E (2016). Autophagy maintains stemness by preventing senescence. Nature.

[158] Thorburn A (2014). Autophagy and its effects: making sense of double-edged swords. PLoS biology.

[159] Ibrahim SA, Gadalla R, El-Ghonaimy EA, Samir O, Mohamed HT, Hassan H (2017). Syndecan-1 is a novel molecular marker for triple negative inflammatory breast cancer and modulates the cancer stem cell phenotype via the IL-6/STAT3, Notch and EGFR signaling pathways. Molecular cancer.

[160] Zeng Z, Fu M, Hu Y, Wei Y, Wei X, Luo M (2023). Regulation and signaling pathways in cancer stem cells: implications for targeted therapy for cancer. Molecular cancer.

[161] Reya T, Clevers H (2005). Wnt signalling in stem cells and cancer. Nature.

[162] Kim SY, Kang JW, Song X, Kim BK, Yoo YD, Kwon YT (2013). Role of the IL-6-JAK1-STAT3-Oct-4 pathway in the conversion of non-stem cancer cells into cancer stem-like cells. Cellular signalling.

[163] Yang Y, Ding L, Hu Q, Xia J, Sun J, Wang X (2017). MicroRNA-218 functions as a tumor suppressor in lung cancer by targeting IL-6/STAT3 and negatively correlates with poor prognosis. Molecular cancer.

[164] Kothari AN, Arffa ML, Chang V, Blackwell RH, Syn WK, Zhang J (2016). Osteopontin-A Master Regulator of Epithelial-Mesenchymal Transition. Journal of clinical medicine.

[165] Blaisdell A, Crequer A, Columbus D, Daikoku T, Mittal K, Dey SK (2015). Neutrophils Oppose Uterine Epithelial Carcinogenesis via Debridement of Hypoxic Tumor Cells. Cancer cell.

[166] Duan Z, Luo Y (2021). Targeting macrophages in cancer immunotherapy. Signal transduction and targeted therapy.

[167] Corliss BA, Azimi MS, Munson JM, Peirce SM, Murfee WL (2016). Macrophages: An Inflammatory Link Between Angiogenesis and Lymphangiogenesis. Microcirculation (New York, NY: 1994).

[168] Cannarile MA, Weisser M, Jacob W, Jegg AM, Ries CH, Rüttinger D (2017). Colony-stimulating factor 1 receptor (CSF1R) inhibitors in cancer therapy. Journal for immunotherapy of cancer.

[169] Shi Q, Shen Q, Liu Y, Shi Y, Huang W, Wang X (2022). Increased glucose metabolism in TAMs fuels O-GlcNAcylation of lysosomal Cathepsin B to promote cancer metastasis and chemoresistance. Cancer cell.

[170] Uribe-Querol E, Rosales C (2015). Neutrophils in Cancer: Two Sides of the Same Coin. Journal of immunology research.

[171] Poillet-Perez L, Despouy G, Delage-Mourroux R, Boyer-Guittaut M (2015). Interplay between ROS and autophagy in cancer cells, from tumor initiation to cancer therapy. Redox biology.

[172] Houghton AM, Rzymkiewicz DM, Ji H, Gregory AD, Egea EE, Metz HE (2010). Neutrophil elastase-mediated degradation of IRS-1 accelerates lung tumor growth. Nature medicine.

[173] Andzinski L, Kasnitz N, Stahnke S, Wu CF, Gereke M, von Köckritz-Blickwede M (2016). Type I IFNs induce anti-tumor polarization of tumor associated neutrophils in mice and human. International journal of cancer.

[174] Dou A, Fang J (2021). Heterogeneous Myeloid Cells in Tumors. Cancers.

[175] Peña-Romero AC, Orenes-Piñero E (2022). Dual Effect of Immune Cells within Tumour Microenvironment: Pro- and Anti-Tumour Effects and Their Triggers. Cancers.

[176] Markowitz GJ, Ban Y, Tavarez DA, Yoffe L, Podaza E, He Y (2024). Deficiency of metabolic regulator PKM2 activates the pentose phosphate pathway and generates TCF1(+) progenitor CD8(+) T cells to improve immunotherapy. Nature immunology.

[177] Kalluri R (2016). The biology and function of fibroblasts in cancer. Nature reviews Cancer.

[178] Sahai E, Astsaturov I, Cukierman E, DeNardo DG, Egeblad M, Evans RM (2020). A framework for advancing our understanding of cancer-associated fibroblasts. Nature reviews Cancer.

[179] Turley SJ, Cremasco V, Astarita JL (2015). Immunological hallmarks of stromal cells in the tumour microenvironment. Nature reviews Immunology.

[180] Fearon DT (2014). The carcinoma-associated fibroblast expressing fibroblast activation protein and escape from immune surveillance. Cancer Immunol Res.

[181] Chang CH, Qiu J, O'Sullivan D, Buck MD, Noguchi T, Curtis JD (2015). Metabolic Competition in the Tumor Microenvironment Is a Driver of Cancer Progression. Cell.

[182] Bertero T, Oldham WM, Grasset EM, Bourget I, Boulter E, Pisano S (2019). Tumor-Stroma Mechanics Coordinate Amino Acid Availability to Sustain Tumor Growth and Malignancy. Cell metabolism.

[183] Sousa CM, Biancur DE, Wang X, Halbrook CJ, Sherman MH, Zhang L (2016). Pancreatic stellate cells support tumour metabolism through autophagic alanine secretion. Nature.

[184] Valkenburg KC, de Groot AE, Pienta KJ (2018). Targeting the tumour stroma to improve cancer therapy. Nature reviews Clinical oncology.

[185] Liu S, Wang X, Sun X, Wei B, Jiang Z, Ouyang Y (2024). Oridonin inhibits bladder cancer survival and immune escape by covalently targeting HK1. Phytomedicine: international journal of phytotherapy and phytopharmacology.

[186] Sun CM, Xiong DB, Yan Y, Geng J, Liu M, Yao XD (2016). Genetic alteration in phosphofructokinase family promotes growth of muscle-invasive bladder cancer. The International journal of biological markers.

[187] Ma Z, Li Z, Mao Y, Ye J, Liu Z, Wang Y (2023). AhR diminishes the efficacy of chemotherapy via suppressing STING dependent type-I interferon in bladder cancer. Nature communications.

[188] Loriot Y, Matsubara N, Park SH, Huddart RA, Burgess EF, Houede N (2023). Erdafitinib or Chemotherapy in Advanced or Metastatic Urothelial Carcinoma. The New England journal of medicine.

[189] Wang D, Mahmud I, Thakur VS, Tan SK, Isom DG, Lombard DB (2024). GPR1 and CMKLR1 Control Lipid Metabolism to Support the Development of Clear Cell Renal Cell Carcinoma. Cancer research.

[190] Sun Q, Xing X, Wang H, Wan K, Fan R, Liu C (2024). SCD1 is the critical signaling hub to mediate metabolic diseases: Mechanism and the development of its inhibitors. Biomedicine & pharmacotherapy = Biomedecine & pharmacotherapie.

[191] Heravi G, Yazdanpanah O, Podgorski I, Matherly LH, Liu W (2022). Lipid metabolism reprogramming in renal cell carcinoma. Cancer metastasis reviews.

[192] Zhou L, Luo Y, Liu Y, Zeng Y, Tong J, Li M (2023). Fatty Acid Oxidation Mediated by Malonyl-CoA Decarboxylase Represses Renal Cell Carcinoma Progression. Cancer research.

[193] He Y, Xu W, Xiao YT, Huang H, Gu D, Ren S (2022). Targeting signaling pathways in prostate cancer: mechanisms and clinical trials. Signal transduction and targeted therapy.

[194] Tolis G, Ackman D, Stellos A, Mehta A, Labrie F, Fazekas AT (1982). Tumor growth inhibition in patients with prostatic carcinoma treated with luteinizing hormone-releasing hormone agonists. Proceedings of the National Academy of Sciences of the United States of America.

[195] Awad D, Cao PHA, Pulliam TL, Spradlin M, Subramani E, Tellman TV (2024). Adipose Triglyceride Lipase Is a Therapeutic Target in Advanced Prostate Cancer That Promotes Metabolic Plasticity. Cancer research.

[196] Dong B, Xu JY, Huang Y, Guo J, Dong Q, Wang Y (2024). Integrative proteogenomic profiling of high-risk prostate cancer samples from Chinese patients indicates metabolic vulnerabilities and diagnostic biomarkers. Nature cancer.

[197] Nirschl CJ, Drake CG (2013). Molecular pathways: coexpression of immune checkpoint molecules: signaling pathways and implications for cancer immunotherapy. Clinical cancer research: an official journal of the American Association for Cancer Research.

[198] Patsoukis N, Bardhan K, Chatterjee P, Sari D, Liu B, Bell LN (2015). PD-1 alters T-cell metabolic reprogramming by inhibiting glycolysis and promoting lipolysis and fatty acid oxidation. Nature communications.

[199] Steggerda SM, Bennett MK, Chen J, Emberley E, Huang T, Janes JR (2017). Inhibition of arginase by CB-1158 blocks myeloid cell-mediated immune suppression in the tumor microenvironment. Journal for immunotherapy of cancer.

[200] Zhao F, Xiao C, Evans KS, Theivanthiran T, DeVito N, Holtzhausen A (2018). Paracrine Wnt5a-β-Catenin Signaling Triggers a Metabolic Program that Drives Dendritic Cell Tolerization. Immunity.

[201] Giovanelli P, Sandoval TA, Cubillos-Ruiz JR (2019). Dendritic Cell Metabolism and Function in Tumors. Trends in immunology.

[202] Yin X, Zeng W, Wu B, Wang L, Wang Z, Tian H (2020). PPARα Inhibition Overcomes Tumor-Derived Exosomal Lipid-Induced Dendritic Cell Dysfunction. Cell reports.

[203] Xu C, Ji X, Zhou Y, Cheng Y, Guo D, Li Q (2023). Slimming and Reinvigorating Tumor-Associated Dendritic Cells with Hierarchical Lipid Rewiring Nanoparticles. Advanced materials (Deerfield Beach, Fla).

[204] McLane LM, Abdel-Hakeem MS, Wherry EJ (2019). CD8 T Cell Exhaustion During Chronic Viral Infection and Cancer. Annual review of immunology.

[205] Peralta RM, Xie B, Lontos K, Nieves-Rosado H, Spahr K, Joshi S (2024). Dysfunction of exhausted T cells is enforced by MCT11-mediated lactate metabolism. Nature immunology.

[206] Guo D, Tong Y, Jiang X, Meng Y, Jiang H, Du L (2022). Aerobic glycolysis promotes tumor immune evasion by hexokinase2-mediated phosphorylation of IκBα. Cell metabolism.

[207] Carr EL, Kelman A, Wu GS, Gopaul R, Senkevitch E, Aghvanyan A (2010). Glutamine uptake and metabolism are coordinately regulated by ERK/MAPK during T lymphocyte activation. Journal of immunology (Baltimore, Md: 1950).

[208] Krall AS, Xu S, Graeber TG, Braas D, Christofk HR (2016). Asparagine promotes cancer cell proliferation through use as an amino acid exchange factor. Nature communications.

[209] Kao KC, Vilbois S, Tsai CH, Ho PC (2022). Metabolic communication in the tumour-immune microenvironment. Nature cell biology.

[210] Herber DL, Cao W, Nefedova Y, Novitskiy SV, Nagaraj S, Tyurin VA (2010). Lipid accumulation and dendritic cell dysfunction in cancer. Nature medicine.

[211] Martin-Perez M, Urdiroz-Urricelqui U, Bigas C, Benitah SA (2022). The role of lipids in cancer progression and metastasis. Cell metabolism.

[212] Xu S, Chaudhary O, Rodríguez-Morales P, Sun X, Chen D, Zappasodi R (2021). Uptake of oxidized lipids by the scavenger receptor CD36 promotes lipid peroxidation and dysfunction in CD8(+) T cells in tumors. Immunity.

[213] Ma X, Bi E, Lu Y, Su P, Huang C, Liu L (2019). Cholesterol Induces CD8(+) T Cell Exhaustion in the Tumor Microenvironment. Cell metabolism.

[214] Soula M, Unlu G, Welch R, Chudnovskiy A, Uygur B, Shah V (2024). Glycosphingolipid synthesis mediates immune evasion in KRAS-driven cancer. Nature.

[215] Nam H, Kundu A, Karki S, Kirkman RL, Chandrashekar DS, Foote JB (2025). HDAC7 promotes renal cancer progression by reprogramming branched-chain amino acid metabolism. Science advances.

[216] Xu Y, Xia Z, Sun X, Wei B, Fu Y, Shi D (2023). Identification of a glutamine metabolism reprogramming signature for predicting prognosis, immunotherapy efficacy, and drug candidates in bladder cancer. Frontiers in immunology.

[217] Liu Z, Xin P, Wu W, Jin M, Du Y, Jiang Y (2025). Nanoparticle-mediated targeting of PGC-1α reveals critical metabolic pathways in bladder cancer metastasis. Communications biology.

[218] Chen Y, Gao M, Chen P, Sharma A, Setiawan MF, Gonzalez-Carmona MA (2025). Comprehensive bioinformatics and immunohistochemical analyses identify phosphoinositide metabolism and PNPLA7 as potential biomarkers in urological cancers. Scientific reports.

[219] Petrella G, Ciufolini G, Vago R, Cicero DO (2021). Urinary Metabolic Markers of Bladder Cancer: A Reflection of the Tumor or the Response of the Body?. Metabolites.

[220] Wang Z, Liu X, Liu X, Sun H, Guo Z, Zheng G (2019). UPLC-MS based urine untargeted metabolomic analyses to differentiate bladder cancer from renal cell carcinoma. BMC cancer.

[221] Lord CJ, Ashworth A (2017). PARP inhibitors: Synthetic lethality in the clinic. Science (New York, NY).

[222] de Bono J, Mateo J, Fizazi K, Saad F, Shore N, Sandhu S (2020). Olaparib for Metastatic Castration-Resistant Prostate Cancer. The New England journal of medicine.

[223] Ali A, Levantini E, Teo JT, Goggi J, Clohessy JG, Wu CS (2018). Fatty acid synthase mediates EGFR palmitoylation in EGFR mutated non-small cell lung cancer. EMBO molecular medicine.

